# Sesquiterpene and Sorbicillinoid Glycosides from the Endophytic Fungus *Trichoderma longibrachiatum* EN-586 Derived from the Marine Red Alga *Laurencia obtusa*

**DOI:** 10.3390/md20030177

**Published:** 2022-02-28

**Authors:** Ying Wang, Xiao-Ming Li, Sui-Qun Yang, Fan-Zhong Zhang, Bin-Gui Wang, Hong-Lei Li, Ling-Hong Meng

**Affiliations:** 1 CAS and Shandong Province Key Laboratory of Experimental Marine Biology, Institute of Oceanology, Chinese Academy of Sciences, Nanhai Road 7, Qingdao 266071, China; wangying194@mails.ucas.ac.cn (Y.W.); lixmqd@qdio.ac.cn (X.-M.L.); suiqunyang@163.com (S.-Q.Y.); fancyzfz@163.com (F.-Z.Z.); wangbg@ms.qdio.ac.cn (B.-G.W.); 2 Laboratory of Marine Biology and Biotechnology, Qingdao National Laboratory for Marine Science and Technology, Wenhai Road 1, Qingdao 266237, China; 3 University of Chinese Academy of Sciences, Yuquan Road 19A, Beijing 100049, China; 4 Center for Ocean Mega-Science, Chinese Academy of Sciences, Nanhai Road 7, Qingdao 266071, China

**Keywords:** *Trichoderma longibrachiatum*, sesquiterpene glycoside, secondary metabolites, antimicrobial activity

## Abstract

An unusual sesquiterpene glycoside trichoacorside A (**1**) and two novel sorbicillinoid glycosides sorbicillisides A (**2**) and B (**3**), together with a known compound sorbicillin (**4**), were isolated and identified from the culture extract of an endophytic fungus *Trichoderma longibrachiatum* EN-586, obtained from the marine red alga *Laurencia obtusa*. Trichoacorside A (**1**) is the first representative of a glucosamine-coupled acorane-type sesquiterpenoid. Their structures were elucidated based on detailed interpretation of NMR and mass spectroscopic data. The absolute configurations were determined by X-ray crystallographic analysis, chemical derivatization, and DP4+ probability analysis. The antimicrobial activities of compounds **1**–**4** against several human, aquatic, and plant pathogens were evaluated.

## 1. Introduction

The prevalence of microbial resistance has become a serious public health threat, highlighting the urgence of screening for new active molecules [1]. Acorane-type sesquiterpenes and sorbicillinoids are common secondary metabolites discovered in several genera of fungi, which displayed various biological activities, including antimicrobial, cytotoxic, anti-inflammatory, and radical-scavenging activities [2,3,4,5]. Though related analogues with unique and diverse structural features have been reported, their glycosides are unusual in natural products research. Marine-derived fungi have shown great potential for structurally unique secondary metabolites with interesting biological and pharmacological properties [6,7], among which algicolous fungi represent an important source of active metabolites [7,8].

The marine red alga *Laurencia obtusa* distributed widely on the coastlines and was used as a traditional medicinal and edible species in China [9,10]. In our ongoing research for bioactive secondary metabolites from marine-derived fungi [11,12,13,14], the endophytic fungus *Trichoderma longibrachiatum* EN-586, which was obtained from the inner tissue of the marine red alga *Laurencia obtusa*, attracted our attention due to its unique secondary metabolite profile. Chemical investigation on the culture extract of *T. longibrachiatum* EN-586 resulted in the isolation and identification of an unusual sesquiterpene glycoside, trichoacorside A (**1**), and two novel sorbicillinoid glycosides, sorbicillisides A (**2**) and B (**3**), together with a known compound sorbicillin (**4**). Herein, we report the chemical investigation of a rice-based culture of *T. longibrachiatum* EN-586 including the isolation, structure elucidation, and biological evaluation of compounds **1**–**4**.

## 2. Results and Discussion

### 2.1. Structure Elucidation

**Figure 1:**
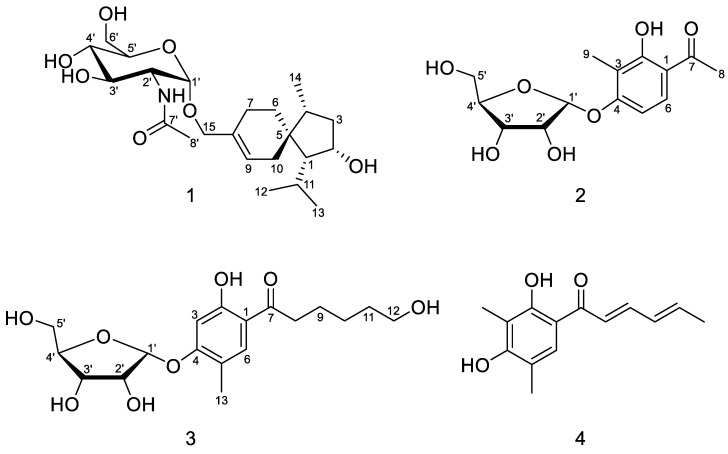
Chemical structures of compounds **1**–**4**.

Isolation and purification of the EtOAc extract of *T. longibrachiatum* EN-586 on solid rice medium by a combination of column chromatography including Lobar LiChroprep RP-18, silica gel, Sephadex LH-20, and semipreparative HPLC, yielded compounds **1**–**4** (Figure 1).

**Figure 2:**
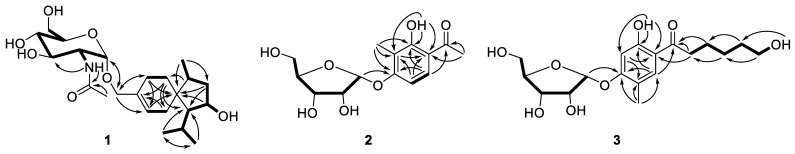
Key HMBC (arrows) and COSY (bold lines) correlations of compounds **1**–**3**.

**Figure 3:**
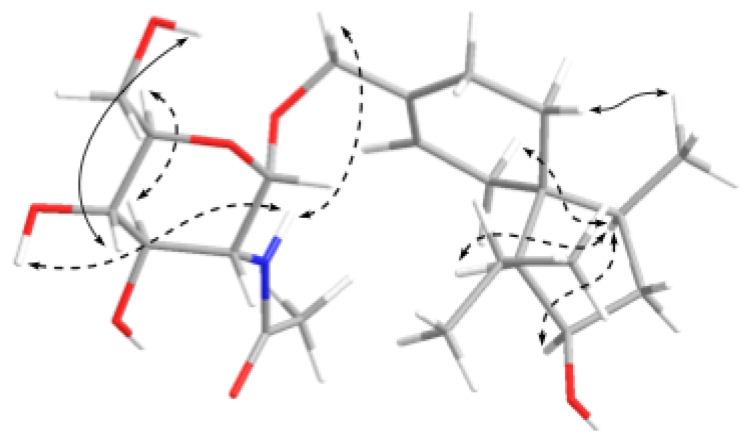
Key NOESY correlations for compound **1**. (Solid line indicates *β*-orientation and dashed line represents *α*-orientation).

Trichoacorside A (**1**) was obtained as yellowish oil and its molecular formula was deduced to be C_23_H_39_NO_7_ by HRESIMS ion peak at *m*/*z* 442.2799 [M + H]^+^ (calcd for C_23_H_40_NO_7_, 442.2799), with five degrees of unsaturation (Figure S7). The ^13^C NMR and DEPT data of **1** (Table 1 and Figure S2) showed 23 carbon signals, containing four methyls, six methylenes (including two oxygenated), 10 methines (including five oxygenated, one nitrogenated, and one olefinic), and three non-protonated carbons (including one olefinic and one carbonyl). Its ^1^H NMR and HSQC spectra (Table 1 and Figures S1 and S4), allowed the assignment of five additional exchangeable protons at *δ*_H_ 4.46, 4.54, 4.80, 5.03, and 7.60. Detailed analysis of the NMR data indicated that compound **1** was a sesquiterpene glycoside. The aglycon was found to be an acorane-type sesquiterpene, which was similar to 2*β*-hydroxytrichoacorenol [3]. However, signals for the hydroxylated methine at C-7 (*δ*_C/H_ 69.0/4.25) in 2*β*-hydroxytrichoacorenol disappeared in those of **1**. Instead, signals for an additional methylene resonating at *δ*_C_ 26.2 and *δ*_H_ 1.38, 1.71 (CH_2_-7) were observed in the NMR spectra of **1**. These data suggested that the aglycon was a dehydroxylated analogue of 2*β*-hydroxytrichoacorenol at C-7, which was confirmed by the COSY correlation between H-6 and H-7 (Figure S3) as well as the HMBC correlations from H-6 to C-8 and C-10 and from H-7 to C-5, C-9, and C-15 (Figure S5a–c). Moreover, resonances of the methyl group at *δ*_C_ 19.4 and *δ*_H_ 1.76 (CH_3_-15) in the NMR spectra of 2*β*-hydroxytrichoacorenol were replaced by an oxygenated methylene resonating at *δ*_C_ 66.6 and *δ*_H_ 4.01 and 4.08 (CH_2_-15) in **1**, accounting for the glycosidic site at C-15, which was further supported by the key HMBC correlation from H-1′ to C-15. The *N*-acetylglucosamine was established by the relevant correlations from H-1′ through H-2′, H-3′, and H-4′ to H-5′, and then to H-6′ and from the proton of 2′-NH to H-2′ in the COSY spectrum and by the key HMBC correlations from H-1′ to C-5′ and from the proton of 2′-NH to C-7′ (Figure 2), and by the identical coupling patterns to the *N*-acetylglucosamine part of deinococcucins A–D [11], as well as by the related NOESY correlations (Figure 3). The chemical shift and coupling data of the anomeric proton at *δ*_H_ 4.65 (1H, d, *J* = 3.4, H-1′) in the ^1^H NMR spectrum were indicative of an *α*-configuration [15,16].

The presence of glucosamine moiety and its absolute configuration of compound **1** was further determined by gas chromatography-mass spectroscopy (GC/MS) analysis of the acidic hydrolysate of **1** derivatized with hexamethyldisilazane (HMDS) and trimethylchlorosilane (TMS-Cl). The derivative of glucosamine from *N*-acetylglucosamine in **1** exhibited the same retention time as that of the authentic D-glucosamine derivative, indicating the D-form glucosamine in **1** (Figure S24) [15].

**Figure 4:**
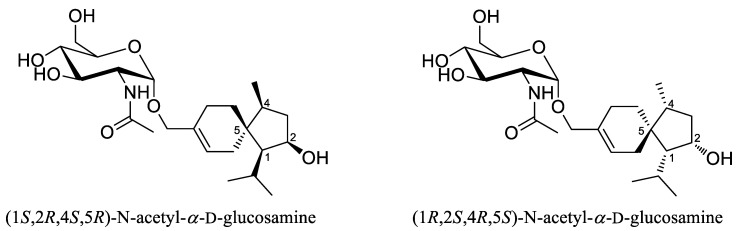
Structures of two possible isomers for DP4+ probability analysis of **1**.

The relative configuration of sesquiterpene moiety was established by analysis of the NOESY spectrum (Figure 3 and Figure S6). The key NOE correlations from H-4 to H-1, H-2, and H-10 oriented these protons toward the same side, while the NOE enhancement from H_3_-14 to H-6 indicated that they were on the opposite side of the molecule. To establish the absolute configuration of the molecule, two possible isomers [(1*S*,2*R*,4*S*,5*R*)-*N*-acetyl-*α*-d-glucosamine and (1*R*,2*S*,4*R*,5*S*)-*N*-acetyl-*α*-d-glucosamine] (Figure 4) were subjected to DP4+ probability analysis. The proton and carbon data of the two possible isomers were calculated based on DP4+ protocol and the results were analyzed with the experimental values [17]. The statistical DP4+ probability analysis of both ^1^H and ^13^C data suggested that the isomer (1*R*,2*S*,4*R*,5*S*)-*N*-acetyl-*α*-d-glucosamine was the equivalent structure with the probability of 100.00% (above 95%) (Figure S25) [17].

Sorbicilliside A (**2**) was originally isolated as a colorless solid. The molecular formula was determined as C_14_H_18_O_7_ according to the HRESIMS ion peak at *m*/*z* 297.0974 [M − H]^−^ (calcd for C_14_H_17_O_7_, 297.0980), implying six unsaturation equivalents (Figure S14). The ^1^H and ^13^C NMR as well as HSQC spectra of **2** (Table 1, Figures S8, S9 and S11) indicated the presence of a ribose moiety, a 1,2,3,4-tetrasubstituted benzene ring, two methyls, and a ketone group as well as an exchangeable proton (*δ*_H_ 12.84). The NMR spectroscopic data displayed signals characteristic of a phenolic glycoside, which were similar to those reported for 4-hydroxy-2-*O*-*α*-ribofuranosyl-5-methylacetophenone [18]. The *α*-ribose moiety was determined by the resonance for the anomeric proton at *δ*_H_ 5.75 (1H, d, *J* = 4.4 Hz, H-1′) and the glycosidic site was established unambiguously by the HMBC correlation from H-1′ (*δ*_H_ 5.75) to C-4 (*δ*_C_ 161.0) [18,19]. The phenolic moiety was established by the COSY correlation of two aromatic methine protons (H-5 and H-6) (Figure S10) as well as the key HMBC correlations from H-5 to C-1 and C-3 and from H-6 to C-2, C-4, and C-7 (Figure 2 and Figure S12). The HMBC correlations from H_3_-8 to C-1 and C-7, from H_3_-9 to C-3 and C-4, and from the proton of 2-OH to C-2 and C-3 assigned the positions of the substituents on the benzene ring (Figure 2). The planar structure of **2** was thus established. Unfortunately, the relative configuration of **2** cannot be assigned as no useful signals observed in the NOESY spectrum (Figure S13).

**Figure 5:**
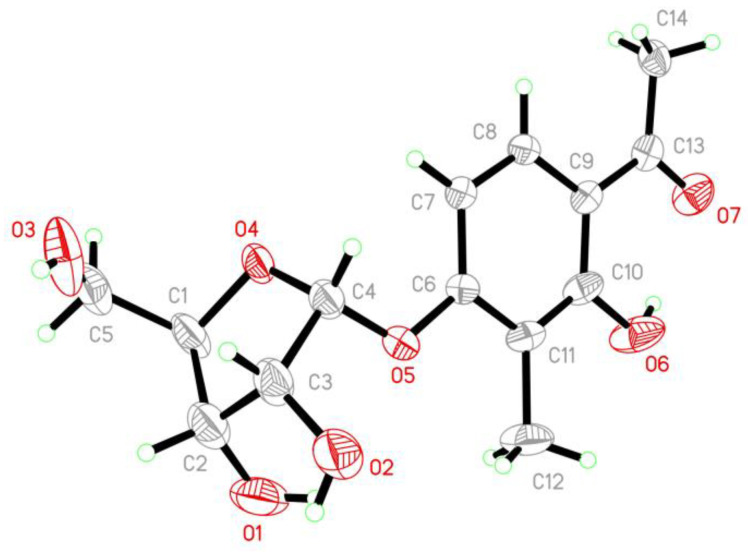
X-ray crystallographic structure of compound **2**.

To clarify the absolute configuration of the ribose moiety, suitable crystals were obtained by dissolving the sample in MeOH-H_2_O (50:1) followed by slow evaporation of the solvents under refrigeration (4 °C). X-ray single crystal diffraction experiment using Cu K*α* radiation (Figure 5) confirmed the structure of **2**. The Flack parameter 0.12(13) allowed for the unambiguous assignment of the ribose moiety as *α*-d-ribose.

Sorbicilliside B (**3**) was isolated as a colorless solid with the molecular formula of C_18_H_26_O_8_ according to the HRESIMS ion peaks at *m*/*z* 371.1711 [M + H]^+^ (calcd for C_18_H_27_O_8_, 371.1700) and 393.1529 [M + Na]^+^ (calcd for C_18_H_26_O_8_Na, 393.1520), accounting for six degrees of unsaturation (Figure S22). The ^1^H and ^13^C NMR spectra of **3** (Table 1, Figures S16 and S17) also showed signals of a ribose moiety, a 1,2,4,5-tetrasubstituted benzene ring, a methyl, five methylenes (including an oxygenated), and a ketone group, as well as two exchangeable protons (*δ*_H_ 4.33 and 12.51). The NMR and ECD data (Figures S16–S21 and S23) of **3** showed resemblance to those of **2** (Figures S8–S13 and S15). However, one of the methyl signals resonating at *δ*_C_ 26.4 and *δ*_H_ 2.58 (CH_3_-8) in **2** were missing in the NMR spectra of **3**, while resonances for five methylenes (with one oxygenated) and an exchangeable proton (*δ*_H_ 4.33) were present in **3** (Table 1), implying the replacement of the methyl in **2** by a pentanol group in **3**. In addition, the methyl substituent on the benzene ring moved from C-3 in **2** to C-5 in **3** as supported by HMBC correlations (Figure 2), resulting in the 1,2,4,5-tetrasubstituted benzene ring of **3**. The planar structure of **3** was further identified by a series of mutually coupled resonances from H-8 through the proton of 12-OH via H-9 through H-12 in the COSY spectrum as well as the key HMBC correlations from H-6 and H-8 to C-7 and from H-1′ to C-4 (Figure 2), with the aglycone part identical to trichosorbicillin G [4]. The absolute configuration of the ribose moiety was further determined by HPLC analysis of the *O*-tolyl isothiocyanate derivative of its acidic hydrolysate [20,21]. The HPLC profiles showed that the product of acidic hydrolysis derivative of compound **3** shared the same retention time as that of *α*-d-ribose derivative (Figure S26).

In addition to a novel acorane-type sesquiterpenoid (**1**) and two new sorbicillinoid glycosides (**2**–**3**), the known compound sorbicillin (**4**), was also isolated and identified by detailed spectroscopic analysis and comparison with the reported data [22].

### 2.2. Antimicrobial Activity

Compounds **1**–**4** were evaluated for their antimicrobial activities against human-, aquatic-, and plant-pathogenic microbes (Table 2). Compound **1** exhibited moderate activity against methicillin-resistant *Staphylococcus aureus*, the aquatic pathogenic bacterium *Vibrio harveyi* as well as most of the tested plant-pathogenic fungi with MIC values ranging from 4 to 64 μg/mL. Compounds **2** and **3** displayed potent activity against *Aeromonas hydrophilia*, both with MIC value of 4 μg/mL, which is comparable to the positive control chloramphenicol (MIC = 2 μg/mL). In addition, compound **4** demonstrated a broad-spectrum of antimicrobial activity against the tested strains with MIC values ranging from 1 to 64 μg/mL. These data indicated that the side chain of the sorbicillinoid glycosides showed a weaker effect on their antimicrobial activities (**2** vs. **3**), while the glycosylation in sorbicillinoid derivatives might increase their activity against the opportunistic pathogen *Aeromonas hydrophilia* (**2** and **3** vs. **4**).

## 3. Materials and Methods

### 3.1. General Experimental Procedures

Column chromatography was performed with commercially available silica gel (200–300 mesh, Qingdao Haiyang Chemical Co., Qingdao, China), Sephadex LH–20 (American Pharmacia) and Lobar LiChroprep RP–18 (40–63 μm, Merck), notably all solvents were used in their anhydrous forms. Thin-layer chromatography (TLC) plates were carried out using precoated silica gel plates GF254 (Qingdao Haiyang Chemical Factory) and analytical HPLC were performed using a Dionex system equipped with P680 pump, ASI-100 automated sample injector, and UVD340U multiple wavelength detector controlled by Chromeleon software (version 6.80). One-dimensional and two-dimensional NMR spectra were determined at 500 MHz for ^1^H and 125 MHz for ^13^C in DMSO-*d*_6_, respectively, on a Bruker Avance 500 spectrometer. Low- or high-resolution ESI mass spectra were recorded on a Waters Micromass Q-TOF Premier and a Thermo Fisher Scientific LTQ Orbitrap XL mass spectrometer. The ECD spectra were measured with CH_3_OH as solvent on a Jasco J-715 spectropolarimeter. Melting points were examined on a SGW X-4 micro-melting-point apparatus. Optical rotations were recorded with a Jasco P-1020 digital polarimeter. UV absorption were evaluated on a Gold S54 Ultraviolet-visible spectrophotometer.

### 3.2. Fungal Material

The fungus *Trichoderma longibrachiatum* EN-586 was obtained from the inner tissue of the marine red alga *Laurencia obtusa* collected from the coast of Qingdao, China in August 2016. The fungal strain was identified based on the morphology and ITS region of the rDNA as described previously [23]. The resulting sequence data *T. longibrachiatum* EN-586 is the same (100%) as that of *T. longibrachiatum* CGAJ1T-2 with accession no. KY495196.1, which has been deposited in GenBank with the accession no. OM060242. The fungus *T. longibrachiatum* EN-586 is preserved at the Key Laboratory of Experimental Marine Biology, Institute of Oceanology of the Chinese Academy of Sciences (IOCAS).

### 3.3. Fermentation, Extraction, and Isolation

The fresh mycelia of *T. longibrachiatum* EN-586 were cultured on PDA medium at 28 °C for 6 days and then inoculated on the rice solid medium in 60 × 1 L conical flasks (each flask contained 70 g rice, 0.1 g corn flour, 0.3 g peptone, and 100 mL natural seawater) for 30 days at room temperature. The whole fermented cultures were repeatedly soaked and extracted for three times with EtOAc, which was evaporated and concentrated under vacuum to obtain a crude extract (28.7 g). 

The extract was fractionated by silica gel vacuum liquid chromatography (VLC) using different solvents of increasing polarity from Petroleum ether (PE)/EtOAc to CH_2_Cl_2_/MeOH to yield ten fractions (Frs. 1–10). Fr. 9 (3.9 g) was fractioned by CC over Lobar LiChroprep RP-18 with a MeOH-H_2_O gradient to yield 10 subfractions (Frs. 9.1–9.10). Fr. 9.4 (156.3 mg) was purified by CC on Sephadex LH-20 (MeOH) and preparative TLC (20 × 20 cm, developing solvents: CH_2_Cl_2_/MeOH 5:1) to obtain compound **1** (3.0 mg). Purification of Fr.4 (2.2 g) with column chromatography (CC) over Lobar LiChroprep RP-18 with a MeOH-H_2_O gradient (from 10:90 to 100) yielded 8 subfractions (Frs. 4.1–4.8). Fr. 4.6 (25.3 mg) was purified by CC on Sephadex LH-20 (MeOH), and then by semipreparative HPLC (Elite ODS-BP column, 10 μm; 20 × 250 mm; 50% MeOH-H_2_O, 8 mL/min) to obtain compound **2** (4.8 mg, *t*_R_23.1 min). Fr.8 (3.8 g) was fractioned by CC over Lobar LiChroprep RP-18 with a MeOH-H_2_O gradient to yield 8 subfractions (Frs. 8.1–8.8). Fr. 8.3 (183.8 mg) was further purified by CC on Sephadex LH-20 (MeOH) and preparative TLC (20 × 20 cm, developing solvents: CH_2_Cl_2_/MeOH 20:1) and then by semipreparative HPLC (Elite ODS-BP column, 10 μm; 20 × 250 mm; 60% MeOH-H_2_O, 8 mL/min) to yield compound **3** (4.9 mg, *t*_R_27.4 min). Fr.8.8 (348.9 mg) was subjected to CC silica gel eluting with PE/EtOAc (4:1) to obtain compound **4** (3.2 mg).

*Trichoacorside A* (**1**): yellowish oil; ${\left[\alpha \right]}_{D}^{25}$ +88.9 (*c* 0.09, MeOH); ^1^H and ^13^C NMR data (Table 1); HRESI-MS *m*/*z* 442.2799 [M + H]^+^, (calcd for C_23_H_40_NO_7_, 442.2799).

*Sorbicilliside A* (**2**): colorless crystals; mp 78–80 °C; ${\left[\alpha \right]}_{D}^{25}$ +100.0 (*c* 0.09, MeOH); ECD (0.67 mM, MeOH) *λ*_max_ (Δ*ε*) 219 (−5.46), 232 (+2.71), 243 (−2.49), 270 (+15.31), 331 (−3.21), 368 (−2.01) nm; UV (MeOH) *λ*_max_ (log *ε*) 216 (3.36), 278 (3.20), 320 (2.64) nm; ^1^H and ^13^C NMR data (Table 1); HRESIMS *m*/*z* 297.0974 [M − H]^−^ (calcd for C_14_H_17_O_7_, 297.0980).

*Sorbicilliside B* (**3**): white solid; ${\left[\alpha \right]}_{D}^{25}$ +100.0 (*c* 0.02, MeOH); ECD (0.54 mM, MeOH) *λ*_max_ (Δ*ε*) 207 (−11.32), 231 (+27.50), 246 (−0.95), 272 (+16.66), 326 (+6.53), 376 (−2.64) nm; UV (MeOH) *λ*_max_ (log *ε*) 213 (3.22), 230 (3.03), 274 (3.08), 327 (2.78) nm; ^1^H and ^13^C NMR data (Table 1); HRESIMS *m*/*z* 371.1700 [M + H]^+^ (calcd for C_18_H_27_O_8_, 371.1711), *m*/*z* 393.1520 [M + Na]^+^ (calcd for C_18_H_26_O_8_Na, 393.1529).

### 3.4. X-ray Crystallographic Analysis of Compound ***2***

By dissolving compound **2** in the solvent of MeOH-H2O (50:1) and storing it in a refrigerator with slow evaporation, suitable crystals were obtained. The crystallographic data [24] were collected over a Bruker D8 Venture CCD diffractometer equipped with graphite-monochromatic Cu-K*α* radiation (*λ* = 1.54178 Å) at 295(2) K. The absorption data were optimized by using the program SADABS [25]. The structures were elucidated strictly with the SHELXTL software package [26,27]. All non-hydrogen atoms were refined anisotropically. The H atoms connected to C atoms were calculated theoretically, and those to O atoms were assigned by difference Fourier maps. The absolute structures were determined by refinement of the Flack parameter [28]. The structures were optimized by full-matrix least-squares techniques.

*Crystal data for compound ***2****: C_14_H_18_O_7_, fw = 298.28, Orthorhombic space group C 2 2 21, unit cell dimensions *a* = 6.9716(8) Å, *b* = 13.646(2) Å, *c* = 32.020(4) Å, *V* = 3046.2(7) Å^3^, α = β = γ = 90°, *Z* = 8, *d*_calcd_ = 1.301 mg/m^3^, crystal dimensions 0.160 × 0.150 × 0.120 mm, *μ* = 0.893 mm^−1^, and *F* (000) = 1264. The 2809 measurements yielded 2265 independent reflections after equivalent data were averaged, and Lorentz and polarization corrections were applied. The final refinement gave *R*_1_ = 0.0719 and w*R*_2_ = 0.2121 [*I* > 2*σ*(*I*)]. The Flack parameter was 0.12(13) in the final refinement for all 2809 reflections with 2265 Friedel pairs.

### 3.5. Acid Hydrolysis and Derivatization of Compound ***1***

The absolute configuration of the glucosamine moiety in compound **1** was determined by the acid hydrolysis with 3 N HCl (0.5 mL) at 80 °C for 2 h to afford sugar moieties and aglycone and after being cooled to room temperature for over 5 hours, the solution mixture was evaporated with the laboratory bench circulating water vacuum pump and then redissolved in pyridine (0.5 mL) with the mixture of hexamethyldisilazane (HMDS) and trimethylchlorosilane (TMS-Cl) (60 µL, *v*/*v* 2:1), furthermore, the solution was heated at 60 °C for 1 hour. The solution was dried with the multifunctional circulating water vacuum pump, the sugar residue was separated with water and CH_2_Cl_2_ (1 mL, *v*/*v* 1:1). The CH_2_Cl_2_ layer was injected into a gas chromatograph based on the previously reported protocol [15,29]. The derivatives of the sugar residue in compound **1** and the authentic d-glucosamine (Solarbio science & technology Co., Ltd, Beijing, China) were analyzed by gas chromatograph-mass (GC, Agilent 7890A/5975C, American, 2012.8) using an HP5 Column (0.25 mm × 30 m × 0.25, Agilent Technologies, Inc., Santa Clara, CA, USA), which was employed with a 41 min temperature program as follows: the initial temperature was maintained at 60 °C for 3 min, ramped to 200 °C at a rate of 4 °C/min, then followed by a 3 min hold at 200 °C. The injector and detector temperatures were maintained at 200 °C, the sample size was controlled at 1 μL and the flow rate of the carrier gas (helium) was 1.0 mL/min, moreover, the split ratio was 10:1. Consequently, the peak of the derivative was detected at 22.486 min (Figure S24), which was identical to the authentic d-glucosamine treated and analyzed using the same protocol, thereby determining the absolute configuration of the glucosamine in **1** as the d-form.

### 3.6. Acid Hydrolysis and Derivatization of Compound ***3***

The absolute configuration of the ribose moiety in compound **3** was established by acid hydrolysis and derivatization. The hydrolyzed sugar fraction (0.5 mg) was dissolved in pyridine (100 μL) containing l-cysteine methyl ester hydrochloride (0.5 mg), incubated at 60 °C for 1 h. A solution of *o*-tolyl isothiocyanate (10 μL) was then added to the mixture and incubated at 60 °C for another 1 h. The mixture was evaporated and dissolved in MeOH to perform reverse-phase HPLC for analysis based on the protocol in the literature [21]. The derivatives of the sugar residue in compound **3** and the authentic d/l-ribose (Aladdin Bio-Chem Technology Co., Ltd., Shanghai, China) were analyzed by analytical HPLC (Elite C18 column, 10 μm; 4.6 × 250 mm;10% acetonitrile-H_2_O for 5 min, then ramped to 100% acetonitrile at a rate of 3%/min, maintained this ratio for 10 min, afterwards, ramped to 10% acetonitrile-H_2_O at a rate of 18%/min, at last, maintained 10% acetonitrile-H_2_O for 10 min; column temperature of 35 °C; flow rate at 1.0 mL/min; detection wavelength at 250 nm), which was equipped with P680 pump, an ASI-100 automated sample injector, a UVD340U multiple wavelength detector controlled by Chromeleon software (version 6.80) and performed on a Dionex HPLC system. The absolute configuration of ribose moiety in compound **3** was determined by comparison of the retention times to those of the authentic derivatives (*t*_R_: d-ribose derivative, 21.865 min, l-ribose derivative, 20.789 min) (Figure S26).

### 3.7. Computational NMR Chemical Shift Calculation and DP4+ Analysis of Compound ***1***

All the theoretical calculations were performed in Gaussian 09 program package. Conformational searches for the possible isomers were carried out through molecular mechanics using the MMFF method with Macromodel software (Schrödinger, LLC., New York, NY, USA) and the corresponding stable conformer, from which distributions higher than 2% were collected. Subsequently, B3LYP/6-31G(d) PCM level in DMSO was used to optimize the conformers. The NMR shielding tensors of all optimized conformers were calculated using the DFT method at mPW1PW91\6-31+G (d) PCM level in DMSO, and then an average based on Boltzmann distribution theory was performed using an equation described previously [17,30]. GIAO (gauge-independent atomic orbital) NMR chemical calculations were conducted using an equation described previously. Finally, the NMR chemical shifts and shielding tensors (^1^H and ^13^C) were analyzed and compared with the experimental chemical shifts using DP4+ probability (Figure S25) [30,31].

### 3.8. Antimicrobial Activity Assay

The antimicrobial activities of the compounds **1**–**4** against the human and aquatic pathogenic bacteria (*Aeromonas hydrophilia* QDIO-1, *Escherichia coli* EMBLC-1, methicillin-resistant *Staphylococcus aureus* (MRSA) EMBLC-4, *Pseudomonas aeruginosa* QDIO-4, *Vibrio harveyi* QDIO-7 and *V. parahaemolyticus* QDIO-8) as well as the plant pathogenic fungi (*Alternaria brassicae* QDIO-11, *Ceratobasidium cornigerum* QDAU-6, *Colletotrichum gloeosporioides* QDAU-31, *C. gloeosporioides Penz*. QDIO-22, *Curvularia spicifera* QDAU-29, *Fusarium graminearum* QDAU-4, *F. oxysporum* QDAU-25, *F. oxysporum* f. sp. *radicis lycopersici* QDAU-10, *F. proliferatum* QDAU-30, *Penicillium digitatum* QDAU-14, and *Physalospora piricola Nose.* QDAU-15) were determined by a serial dilution technique using 96-well microtiter plates as previously reported [32]. Amphotericin B was used as a positive control for fungi, while chloramphenicol as a positive control for bacteria. The human and aquatic pathogenic bacteria and plant pathogenic fungi were offered by the Institute of Oceanology, Chinese Academy of Sciences.

## 4. Conclusions

In summary, we isolated and identified three new glycoside compounds (**1**–**3**) from the marine red alga endophytic fungus *Trichoderma longibrachiatum* EN-586. It is noteworthy that compound **1** represents an unprecedented acorane-type sesquiterpenoid coupled to glucosamine. Compounds **2** and **3** may prove useful as antibiotic agents against the opportunistic pathogen *Aeromonas hydrophilia.*

## Figures and Tables

**Table 1:** ^1^H and ^13^C NMR data for compounds **1**–**3** in DMSO-*d*_6_^a^.

No.	1		2		3	
	*δ*_H_ (*J* in Hz)	*δ*_C_	*δ*_H_ (*J* in Hz)	*δ*_C_	*δ*_H_ (*J* in Hz)	*δ*_C_
1	1.24, overlap	59.3, CH		113.9, C		113.2, C
2	4.22, brs	64.6, CH		161.2, C		162.0, C
3	*α* 1.12, dd (11.4, 3.7) *β* 1.67, m	28.5, CH_2_		113.9, C	6.59, s	102.1, CH
4	1.55, m	46.2, CH		161.0, C		161.3, C
5		44.2, C	6.75, d (9.0)	106.2, CH		118.5, C
6	*α* 1.19, m *β* 1.63, m	31.8, CH_2_	7.77, d (9.0)	130.2, CH	7.71, s	131.8, CH
7	*α* 1.38, m *β* 1.71, overlap	26.2, CH_2_		203.9, C		205.3, C
8		137.3, C	2.58, s	26.4, CH_3_	2.97, t (7.3)	37.6, CH_2_
9	5.69, brs	124.1, CH	2.05, s	7.8, CH_3_	1.62, m	24.1, CH_2_
10	*α* 1.79, overlap *β* 2.07, dt (18.9, 3.0)	34.5, CH_2_			1.35, m	25.2, CH_2_
11	1.61, m	29.8, CH			1.45, m	32.3, CH_2_
12	0.88, d (6.5)	23.3, CH_3_			3.39, dt (6.3, 11.5)	60.6, CH_2_
13	0.83, d (6.5)	22.8, CH_3_			2.13, s	15.3, CH_3_
14	0.80, d (6.7)	14.2, CH_3_				
15	*α* 4.08, d (13.0) *β* 4.01, d (13.0)	66.6, CH_2_				
2-OH	4.54, d (6.0)		12.84, s		12.51, s	
12-OH					4.33, t (5.1)	
1′	4.65, d (3.4)	96.1, CH	5.75, d (4.4)	99.9, CH	5.71, d (4.4)	99.7, CH
2′	3.65, td (8.1, 3.4)	53.9, CH	4.11, m	71.4, CH	4.11, m	71.4, CH
3′	3.45, dd (10.9, 8.1)	70.8, CH	3.96, overlap	69.1, CH	3.96, overlap	69.1, CH
4′	3.15, d (9.0)	70.6, CH	3.98, overlap	86.5, CH	3.96, overlap	86.5, CH
5′	3.39, ddd (10.0, 5.4, 2.3)	72.9, CH	3.47, d (3.6)	61.4, CH_2_	3.47, dd (3.6, 4.9)	61.4, CH_2_
6′	*α* 3.49, overlapped *β* 3.60, m	60.8, CH_2_				
7′		169.3, C				
8′	1.82, s	22.6, CH_3_				
2′-OH			4.61, brs		4.56, d (8.7)	
2′-NH	7.60, d (8.1)					
3′-OH	4.80, brs		4.95, brs		4.92, d (4.6)	
4′-OH	5.03, brs					
5′-OH			4.80, brs		4.81, t (4.9)	
6′-OH	4.46, dd (11.8, 3.5)					

^a 1^H and ^13^C data were recorded at 500 and 125 MHz, respectively.

**Table 2:** Antimicrobial activities of compounds **1**–**4** (MIC, μg/mL) ^a^.

Strains	Compounds				
	1	2	3	4	Positive Control
*A. brassicae* ^b^	32	-	-	16	0.5
*C. cornigerum* ^b^	64	-	-	64	0.5
*C. gloeosporioides* ^b^	16	64	32	-	0.5
*C. gloeosporioides Penz* ^b^	16	32	32	2	0.5
*C. spicifera* ^b^	8	16	8	2	0.25
*F. graminearum* ^b^	-	-	-	32	0.5
*F. oxysporum* ^b^	32	32	32	1	0.5
*F. oxysporum* f. sp. *radicis lycopersici* ^b^	32	-	64	32	0.5
*F. proliferatum* ^b^	32	64	64	2	0.5
*P. digitatum* ^b^	64	32	32	16	0.5
*P. piricola Nose* ^b^	32	-	64	2	0.5
*A. hydrophilia* ^c^	64	4	4	8	2
*E. coli* ^c^	-	64	16	16	1
methicillin-resistant *S. aureus* ^c^	64	-	-	16	8
*P. aeruginosa* ^c^	-	-	-	16	1
*V. harveyi* ^c^	4	16	16	16	0.5
*V. parahaemolyticus* ^c^	-	-	-	4	0.5

^a^ (-) = MIC > 64 μg/mL. ^b^ Amphotericin B as positive control. ^c^ Chloramphenicol as positive control.

## Data Availability

Not applicable.

## Supplementary Materials

The following supporting information can be downloaded at: https://www.mdpi.com/article/10.3390/md20030177/s1, Figure S1: ^1^H NMR (500 MHz, DMSO-*d*_6_) spectrum of compound **1**; Figure S2: ^13^C NMR (125 MHz, DMSO-*d*_6_) and DEPT spectra of compound **1**; Figure S3: COSY spectrum of compound **1**; Figure S4: HSQC spectrum of compound **1**; Figure S5a: HMBC spectrum of compound **1**; Figure S5b: Enlarged HMBC spectrum of compound **1** (lower field); Figure S5c: Enlarged HMBC spectrum of compound **1** (higher field); Figure S6: NOESY spectrum of compound **1**; Figure S7: HRESIMS spectrum of compound **1**; Figure S8: ^1^H NMR (500 MHz, DMSO-*d*_6_) spectrum of compound **2**; Figure S9: ^13^C NMR (125 MHz, DMSO-*d*_6_) and DEPT spectra of compound **2**; Figure S10: COSY spectrum of compound **2**; Figure S11: HSQC spectrum of compound **2**; Figure S12: HMBC spectrum of compound **2**; Figure S13: NOESY spectrum of compound **2**; Figure S14: HRESIMS spectrum of compound **2**; Figure S15: ECD spectrum of compound **2**; Figure S16: ^1^H NMR (500 MHz, DMSO-*d*_6_) spectrum of compound **3**; Figure S17: ^13^C NMR (125 MHz, DMSO-*d*_6_) and DEPT spectra of compound **3**; Figure S18: COSY spectrum of compound **3**; Figure S19: HSQC spectrum of compound **3**; Figure S20: HMBC spectrum of compound **3**; Figure S21: NOESY spectrum of compound **3**; Figure S22: HRESIMS spectrum of compound **3**; Figure S23: ECD spectrum of compound **3**; Figure S24: Determination of absolute configuration of glucosamine in compound **1**; Figure S25: DP4+ probability Excel sheets of compound **1**; Figure S26: Determination of absolute configuration of ribose in compound **3**.
