# Supplementary material for: Sesquiterpene and Sorbicillinoid Glycosides from the Endophytic Fungus Trichoderma longibrachiatum EN-586 Derived from the Marine Red Alga Laurencia obtusa
Source: Mar Drugs. 2022 Feb 28;20(3):177. doi: 10.3390/md20030177 (PMC8949086; doi:10.3390/md20030177)
Supplement: Supplementary file 1 [file marinedrugs-20-00177-s001.zip › marinedrugs-1620815-supplementary.pdf]

Supporting Information

Sesquiterpene and Sorbicillinoid Glycosides from the Endophytic Fungus *Trichoderma longibrachiatum* EN-586 Derived from the Marine Red Alga *Laurencia obtusa*

Ying Wang,[†,‡,§] Xiao-Ming Li,[†,‡] Sui-Qun Yang,[†,‡] Fan-Zhong Zhang,[†,‡] Bin-Gui Wang,[†,‡,§,⊥] Hong-Lei Li,*[,†,‡] and Ling-Hong Meng,*[,†,‡,§]

[†]CAS and Shandong Province Key Laboratory of Experimental Marine Biology, Institute of Oceanology, Chinese Academy of Sciences, Nanhai Road 7, Qingdao 266071, People's Republic of China

[‡]Laboratory of Marine Biology and Biotechnology, Qingdao National Laboratory for Marine Science and Technology, Wenhai Road 1, Qingdao 266237, People's Republic of China

[§]University of Chinese Academy of Sciences, Yuquan Road 19A, Beijing 100049, People's Republic of China

[⊥]Center for Ocean Mega-Science, Chinese Academy of Sciences, Nanhai Road 7, Qingdao 266071, People's Republic of China

Corresponding Authors

*Phone (H.-L. Li): +86-532-82898890. E-mail: lihonglei@qdio.ac.cn.

*Phone (L.-H. Meng): +86-532-82898890. E-mail: menglh@ms.qdio.ac.cn.

Contents

Figure S1. ^{1}H NMR (500 MHz, DMSO-d_6) spectrum of compound **1**;

Figure S2. ^{13}C NMR (125 MHz, DMSO-d_6) and DEPT spectra of compound **1**;

Figure S3. COSY spectrum of compound **1**;

Figure S4. HSQC spectrum of compound **1**;

Figure S5a. HMBC spectrum of compound **1**;

Figure S5b. Enlarged HMBC spectrum of compound **1** (lower field);

Figure S5c. Enlarged HMBC spectrum of compound **1** (higher field);

Figure S6. NOESY spectrum of compound **1**;

Figure S7. HRESIMS spectrum of compound **1**;

Figure S8. ^{1}H NMR (500 MHz, DMSO-d_6) spectrum of compound **2**;

Figure S9. ^{13}C NMR (125 MHz, DMSO-d_6) and DEPT spectra of compound **2**;

Figure S10. COSY spectrum of compound **2**;

Figure S11. HSQC spectrum of compound **2**;

Figure S12. HMBC spectrum of compound **2**;

Figure S13. NOESY spectrum of compound **2**;

Figure S14. HRESIMS spectrum of compound **2**;

Figure S15. ECD spectrum of compound **2**;

Figure S16. ^{1}H NMR (500 MHz, DMSO-d_6) spectrum of compound **3**;

Figure S17. ^{13}C NMR (125 MHz, DMSO-d_6) and DEPT spectra of compound **3**;

Figure S18. COSY spectrum of compound **3**;

Figure S19. HSQC spectrum of compound **3**;

Figure S20. HMBC spectrum of compound **3**;

Figure S21. NOESY spectrum of compound **3**;

Figure S22. HRESIMS spectrum of compound **3**;

Figure S23. ECD spectrum of compound **3**;

Figure S24. Determination of absolute configuration of glucosamine in compound **1**;

Figure S25. DP4+ probability Excel sheets of compound **1**;

Figure S26. Determination of absolute configuration of ribose in compound **3**;

Figure S1. ^{1}H NMR (500 MHz, DMSO-d_6) spectrum of compound **1**;

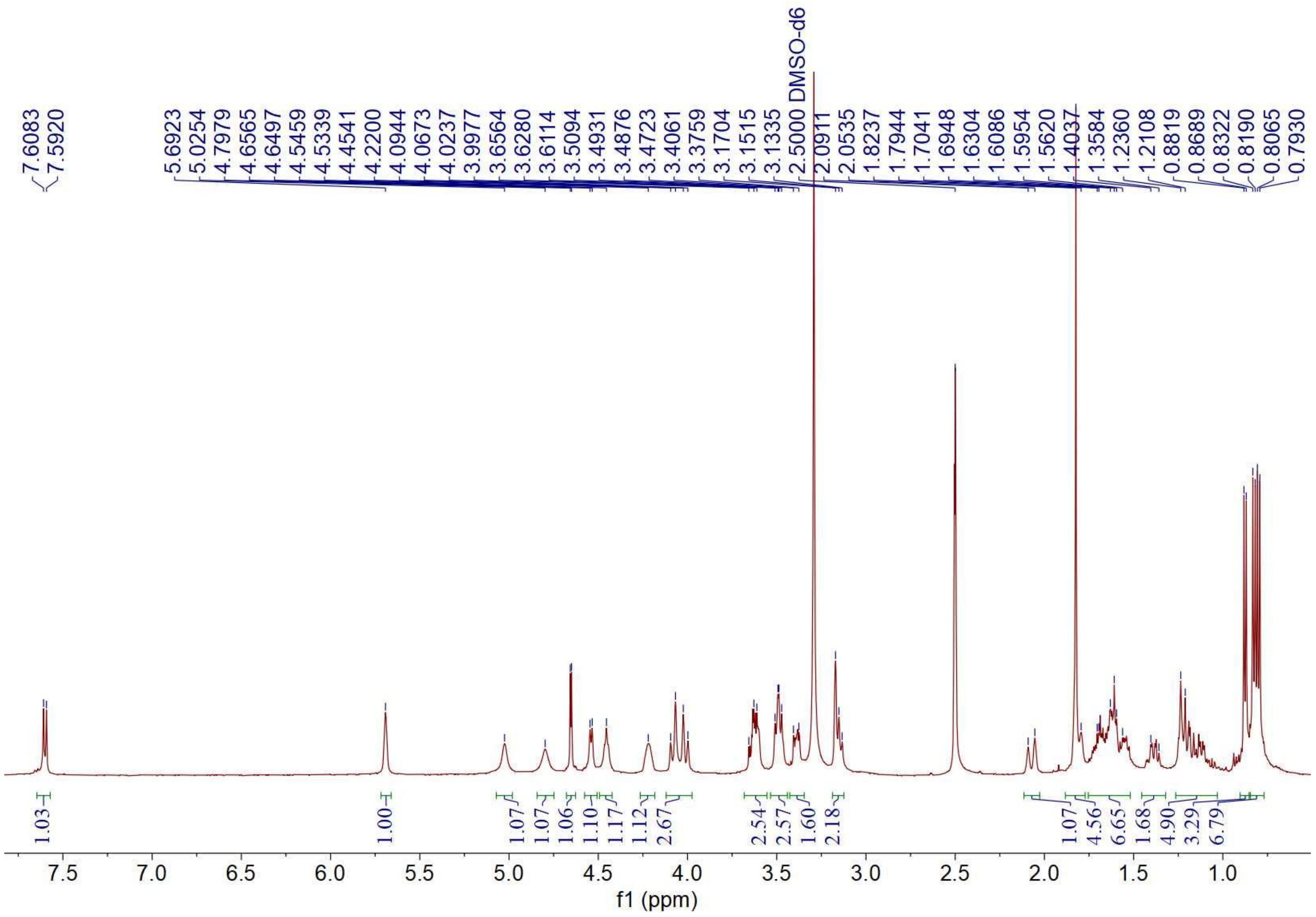

Figure S2. ^{13}C NMR (125 MHz, DMSO-d_6) and DEPT spectra of compound **1**;

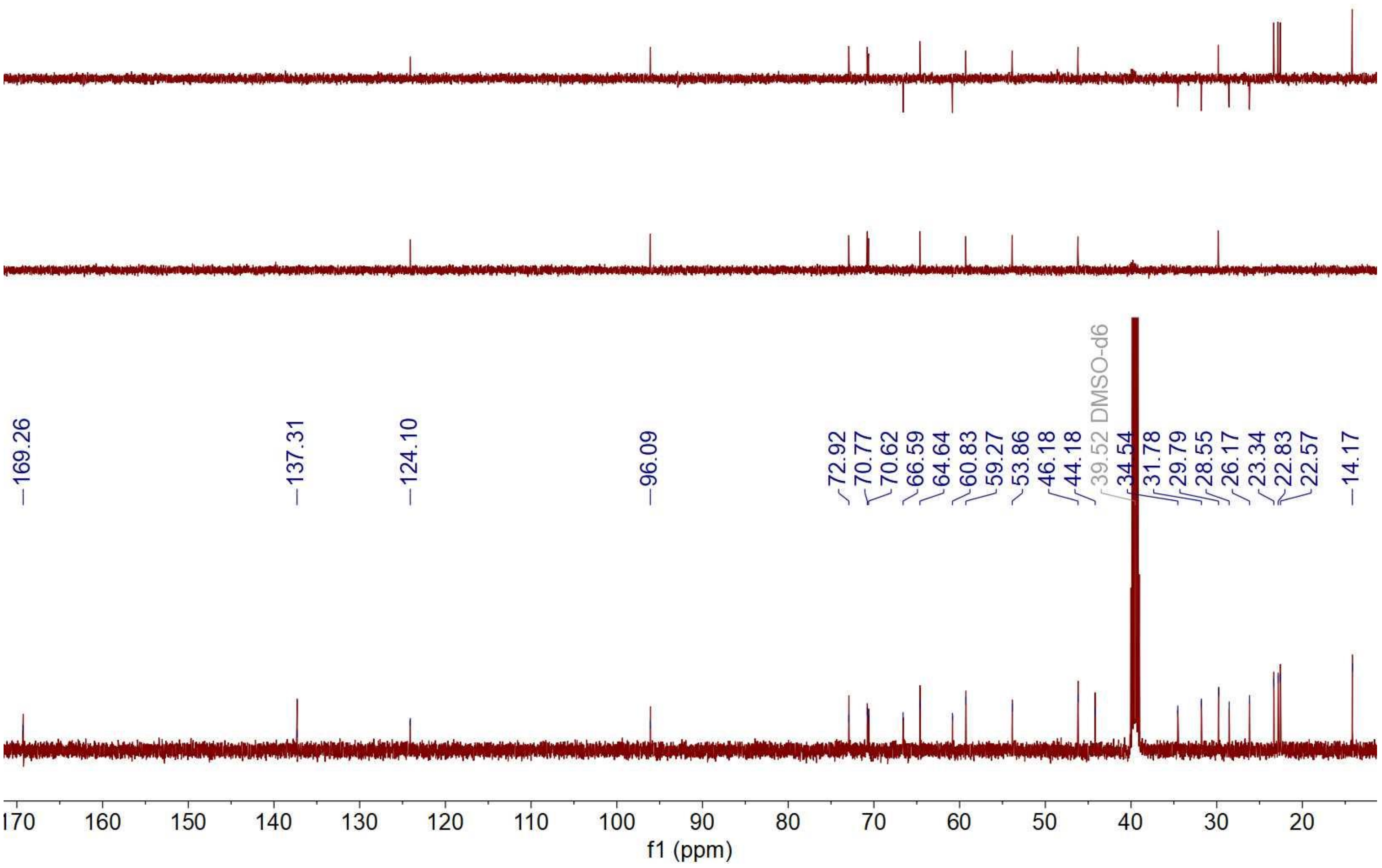

Figure S3. COSY spectrum of compound **1**;

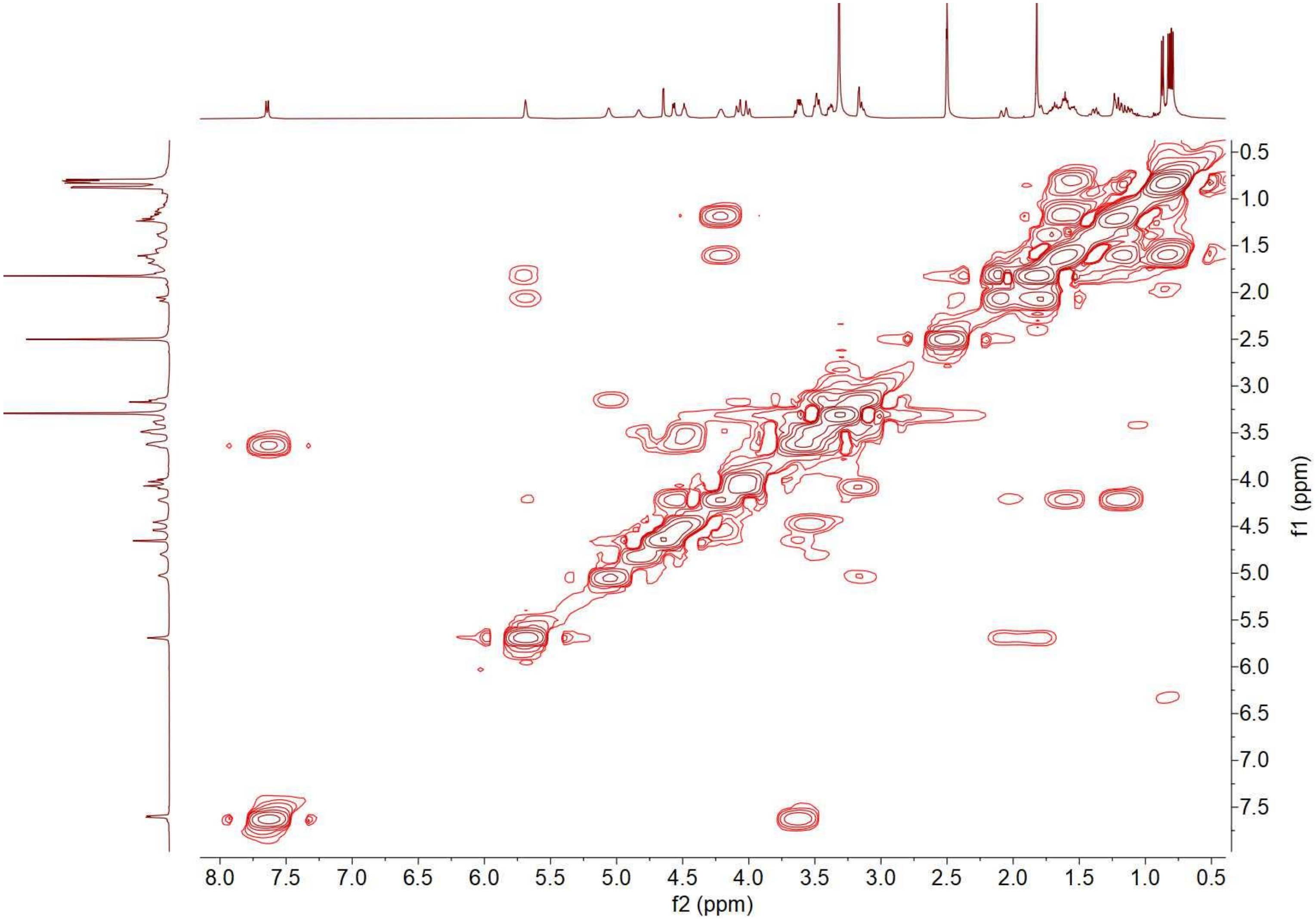

Figure S4. HSQC spectrum of compound **1**;

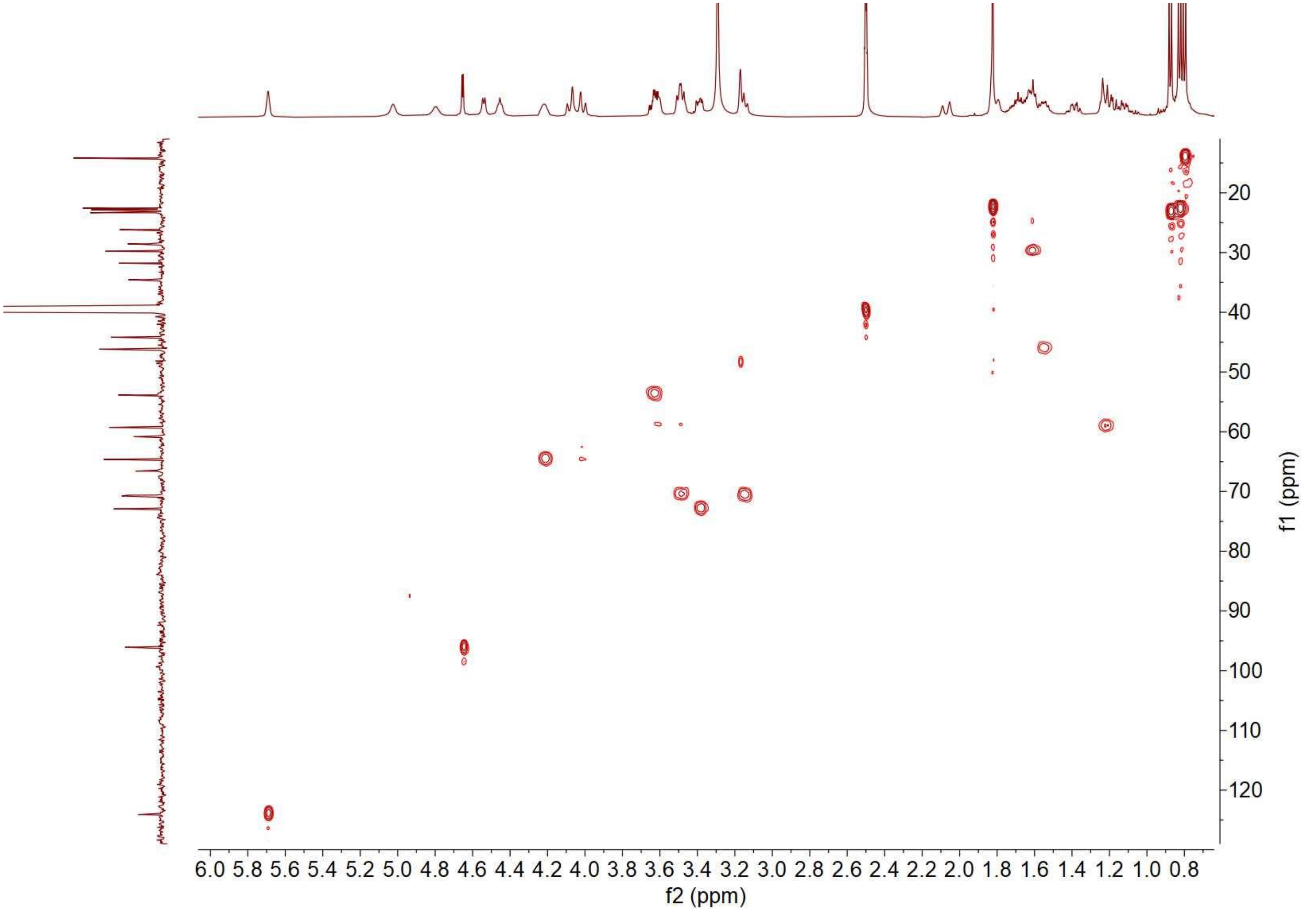

Figure S5a. HMBC spectrum of compound **1**;

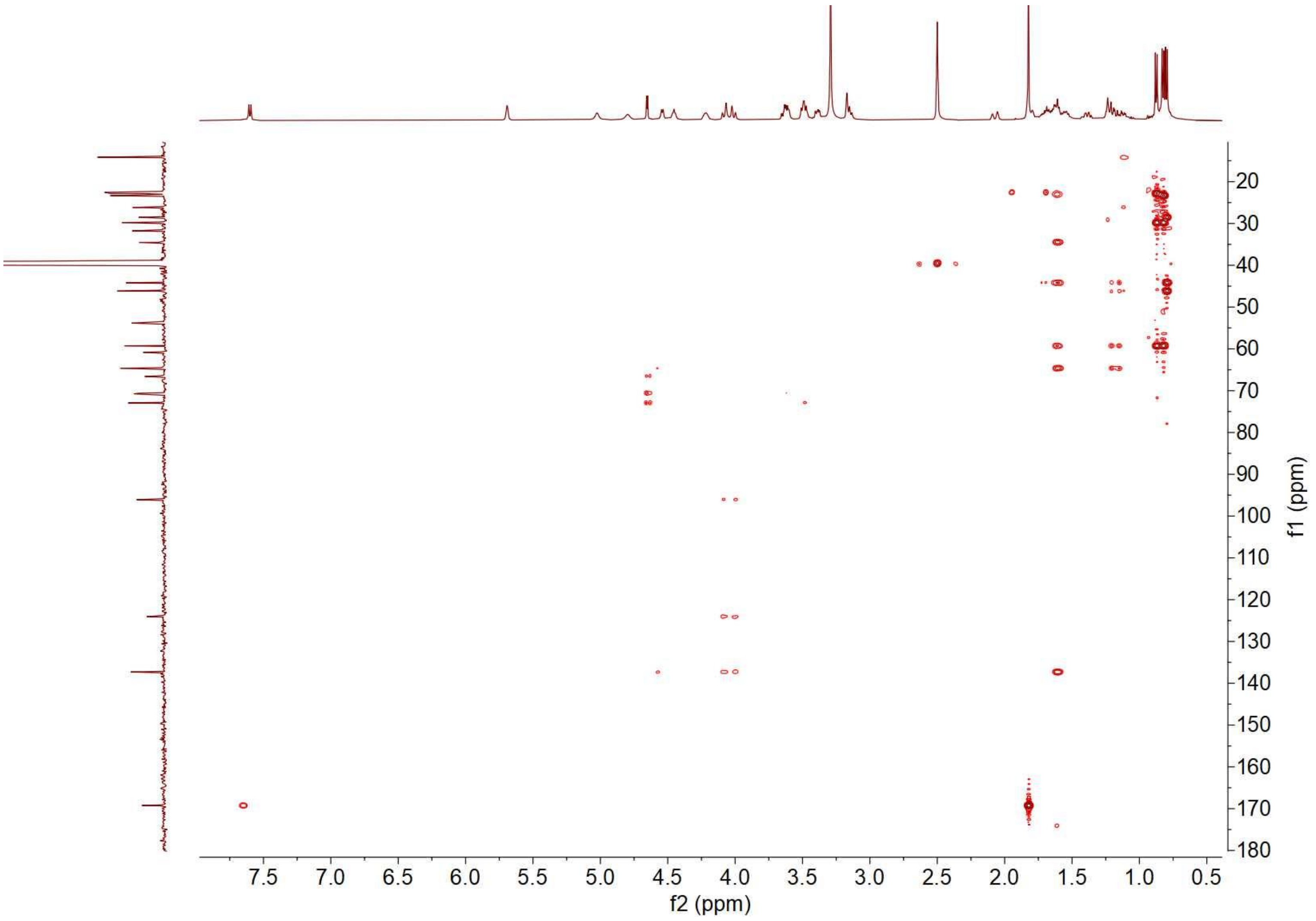

Figure S5b. Enlarged HMBC spectrum of compound **1** (lower field);

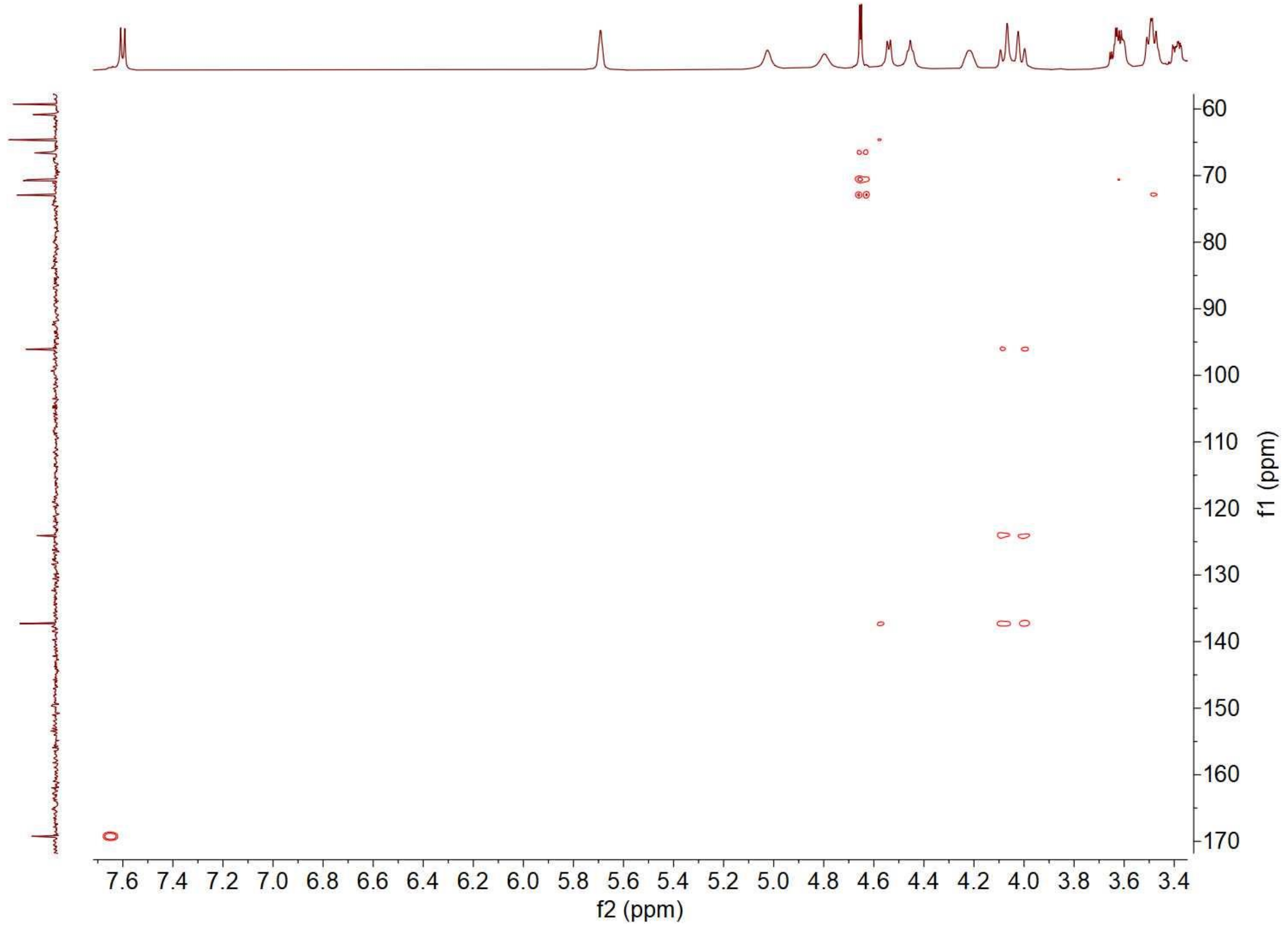

Figure S5c. Enlarged HMBC spectrum of compound **1** (higher field);

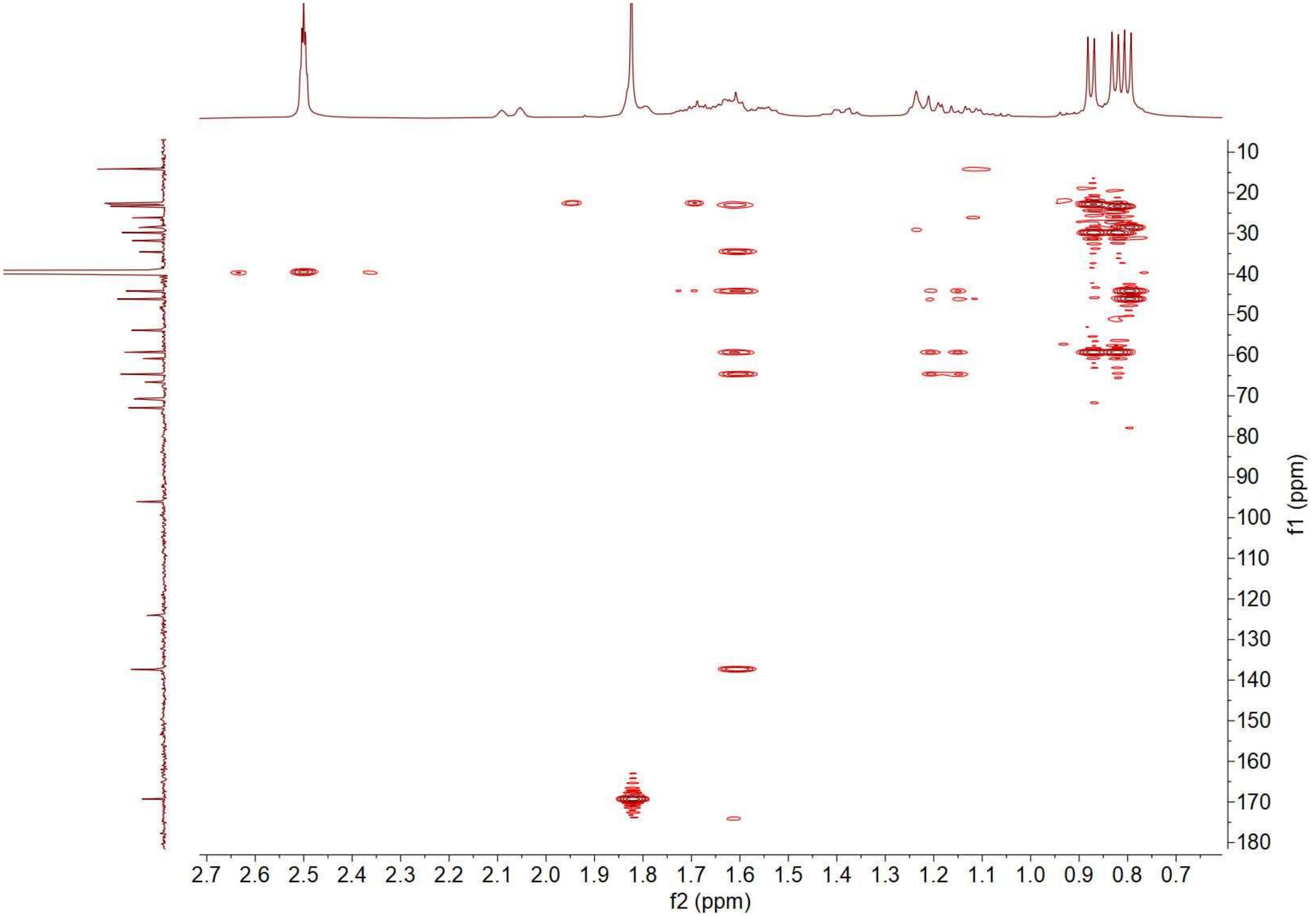

Figure S6. NOESY spectrum of compound **1**;

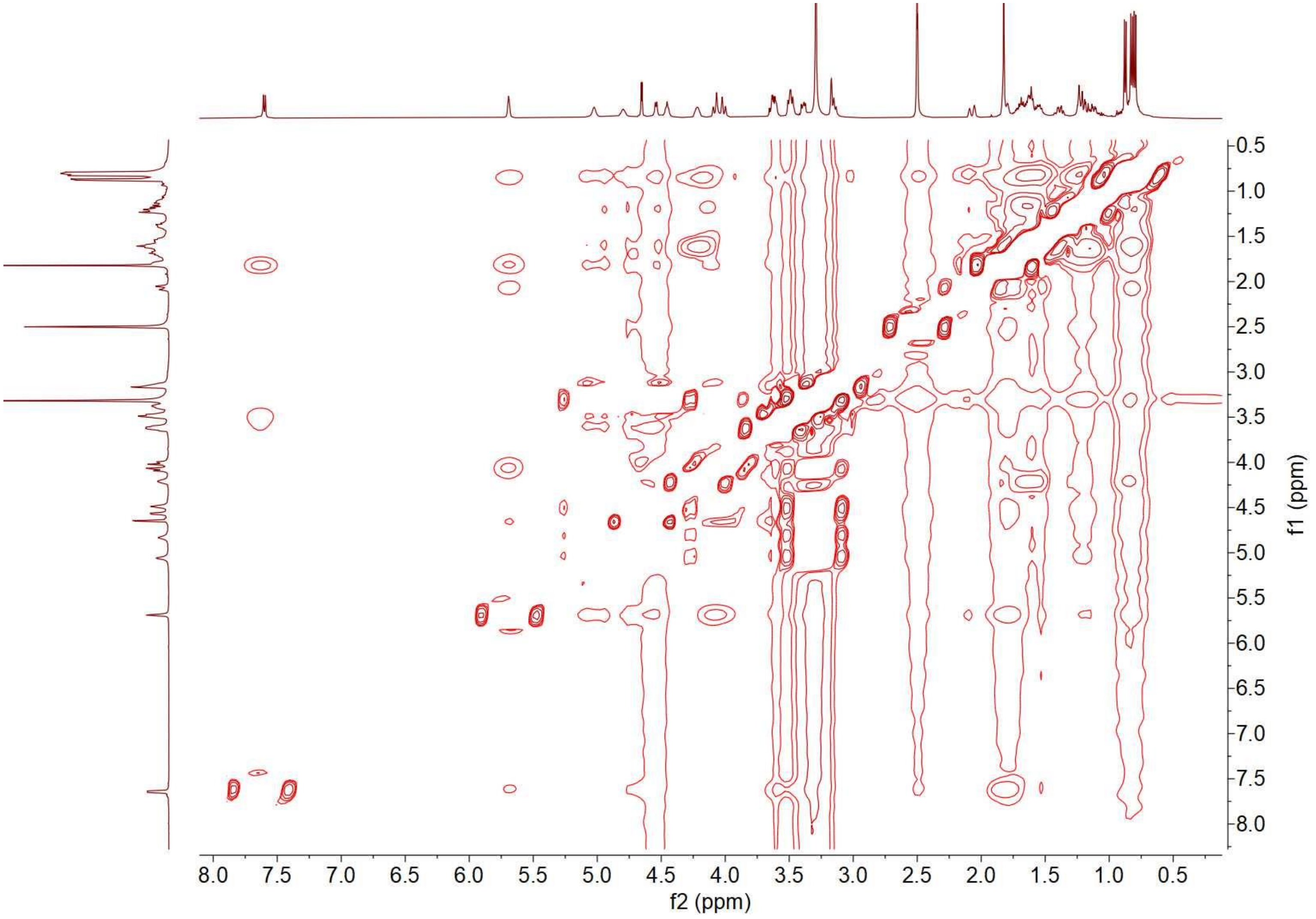

Figure S7. HRESIMS spectrum of compound **1**;

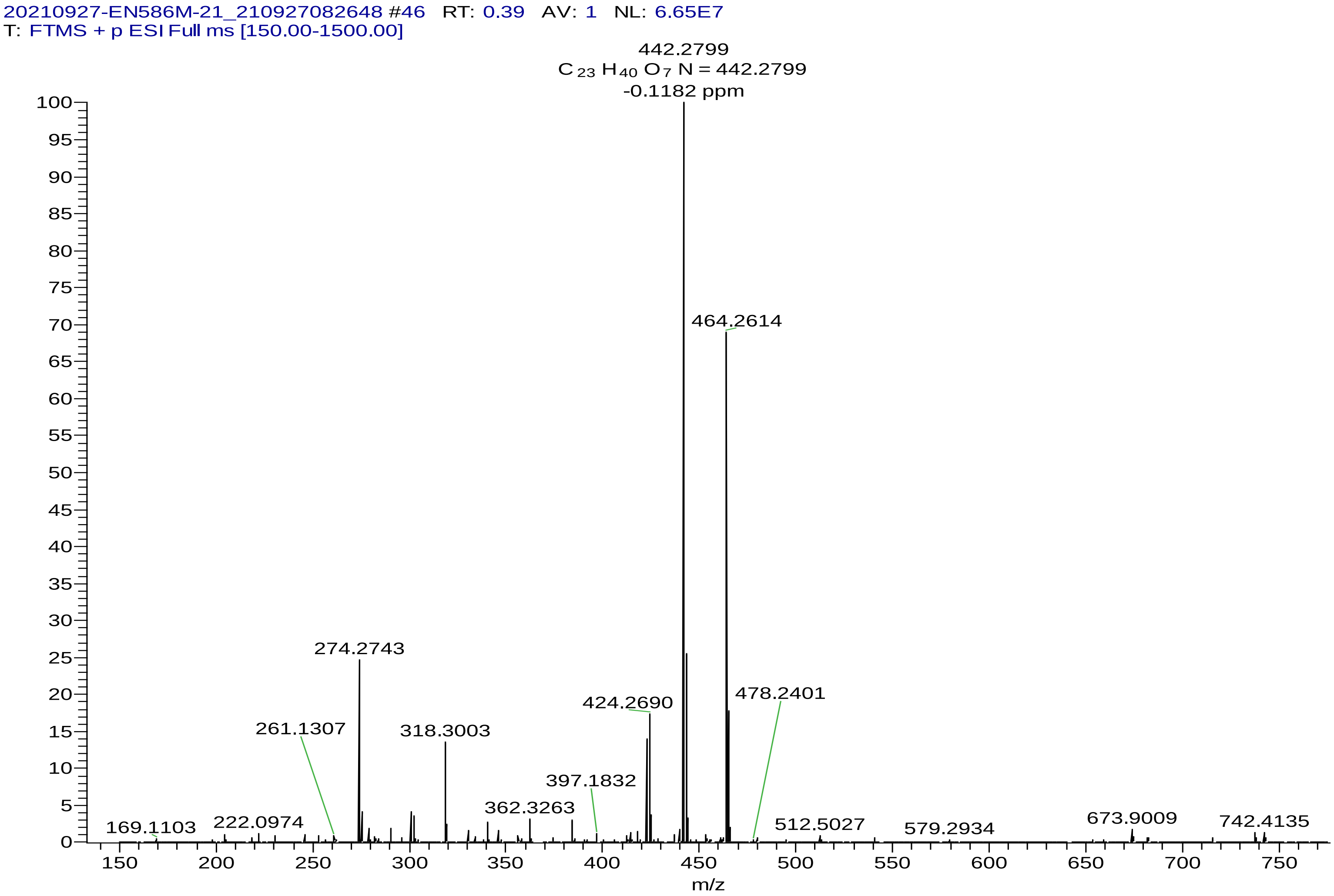

Figure S8. ^{1}H NMR (500 MHz, DMSO-d_6) spectrum of compound **2**;

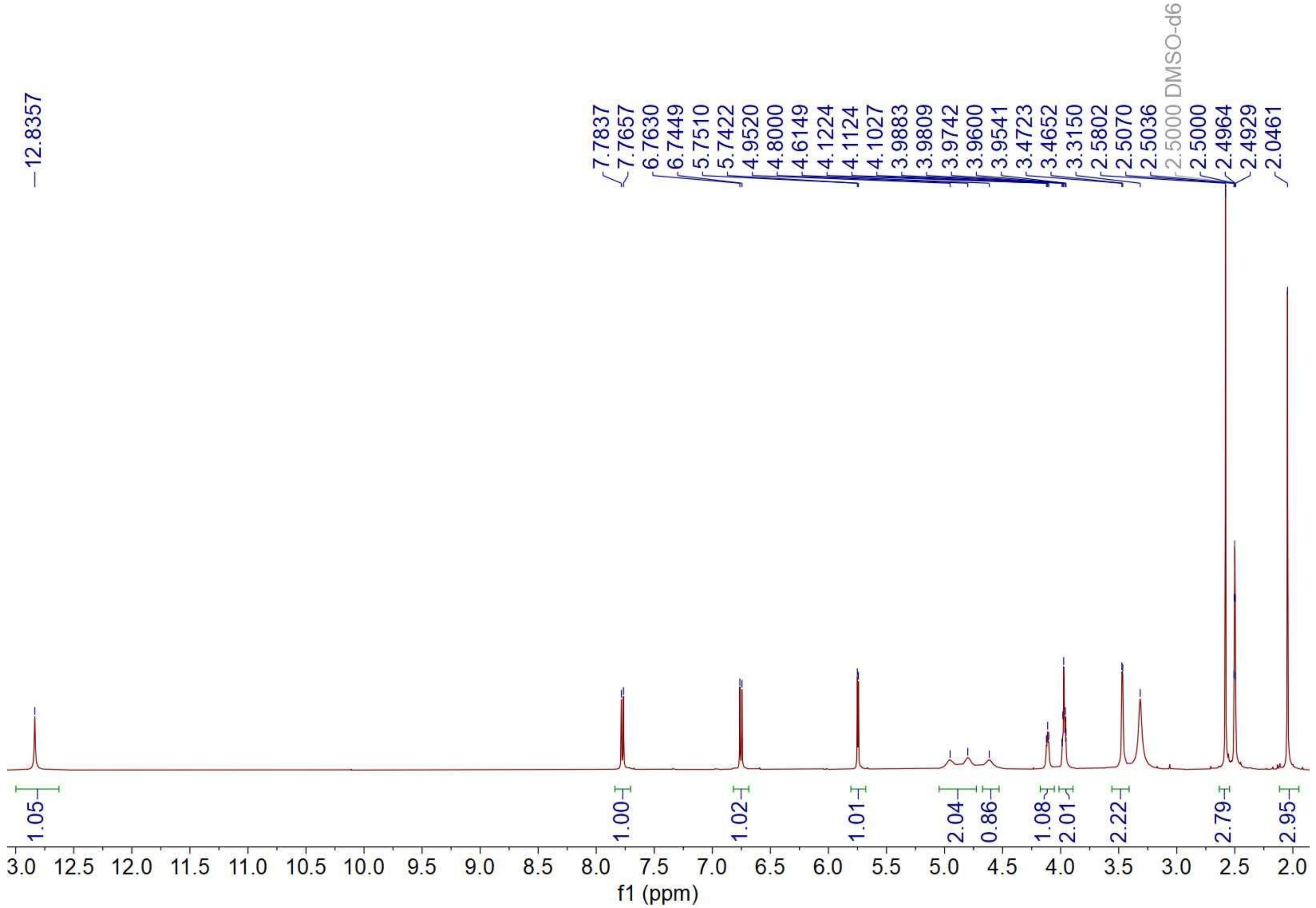

Figure S9. ^{13}C NMR (125 MHz, DMSO-d_6) and DEPT spectra of compound **2**;

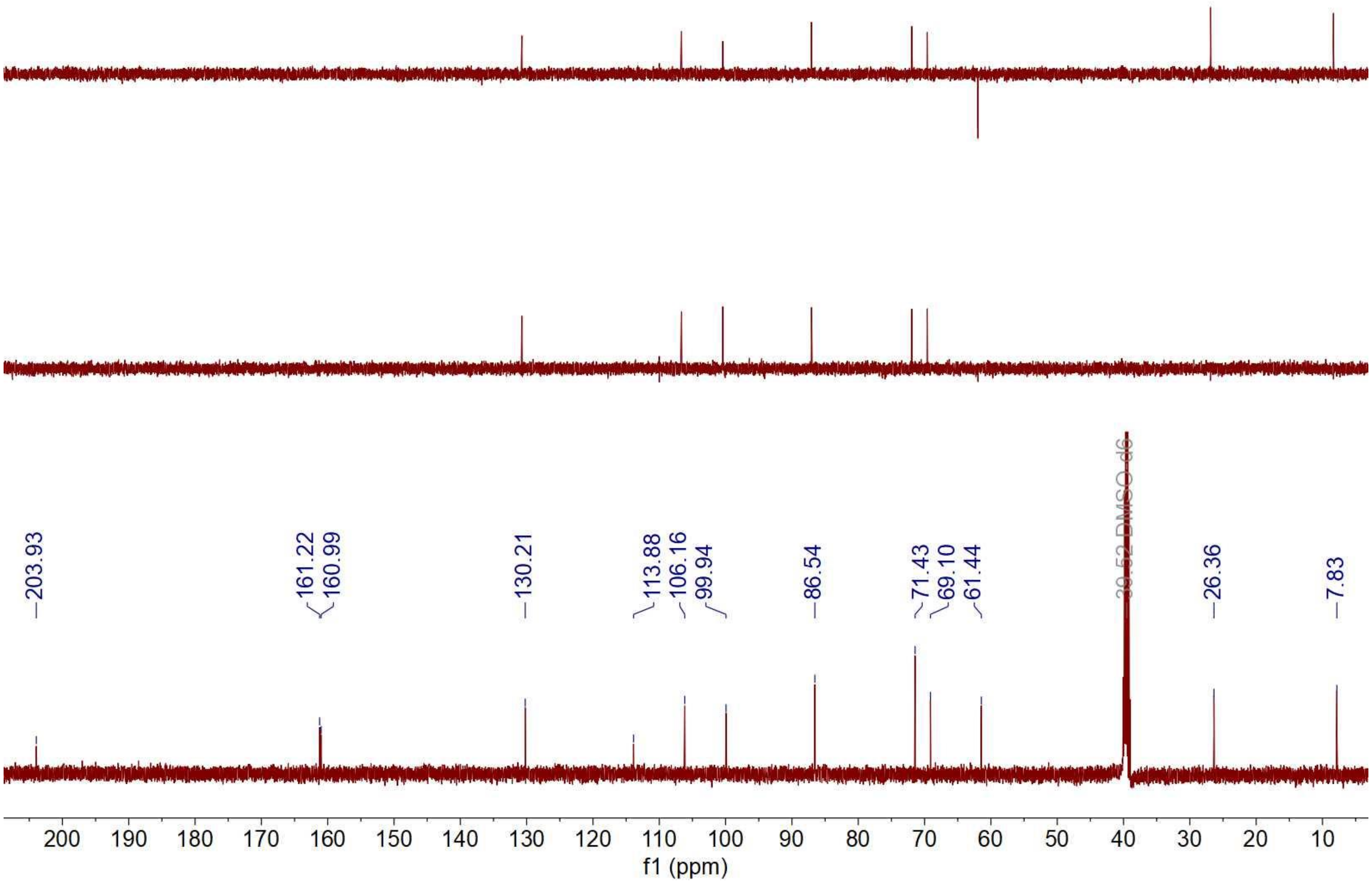

Figure S10. COSY spectrum of compound **2**;

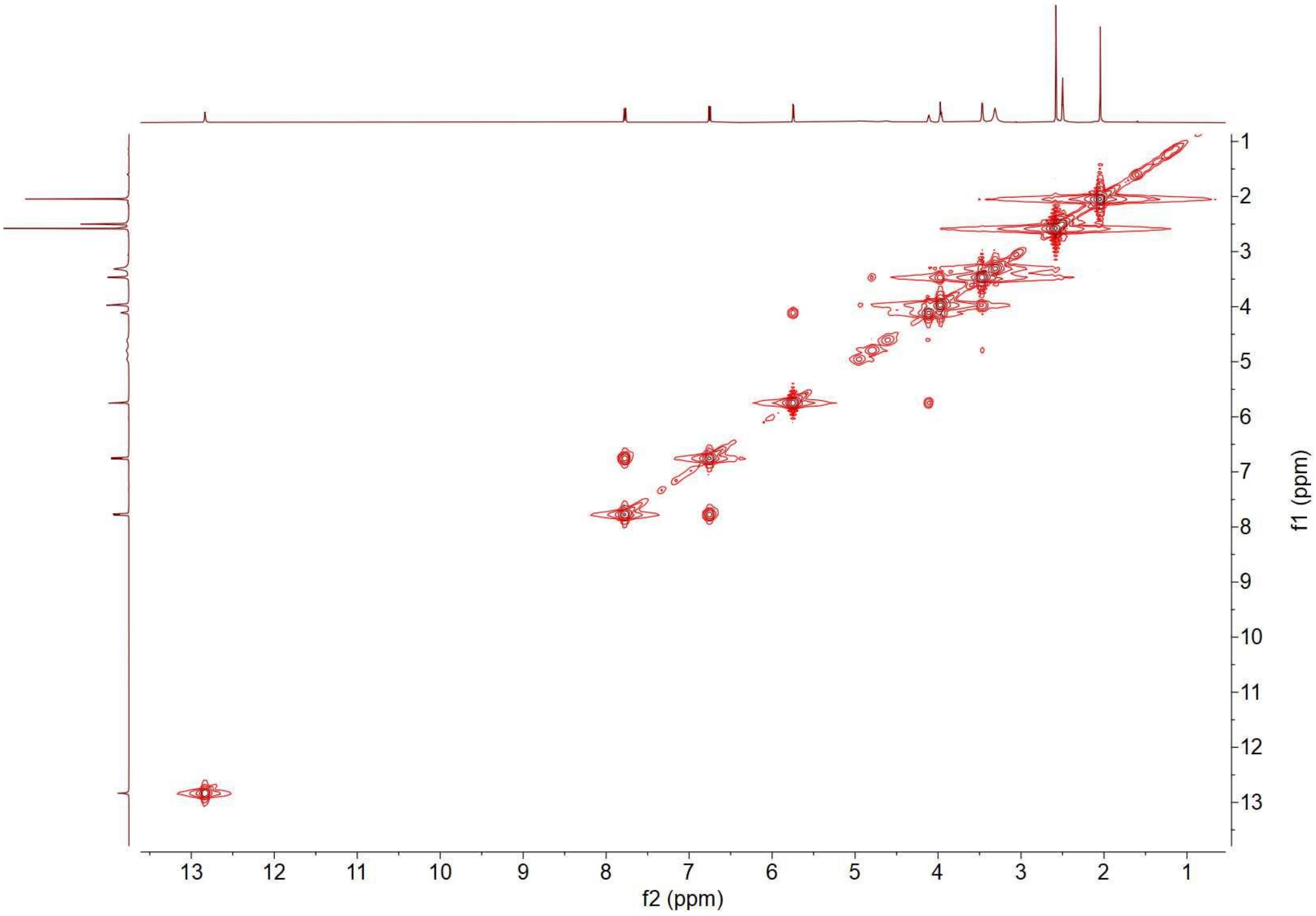

Figure S11. HSQC spectrum of compound **2**;

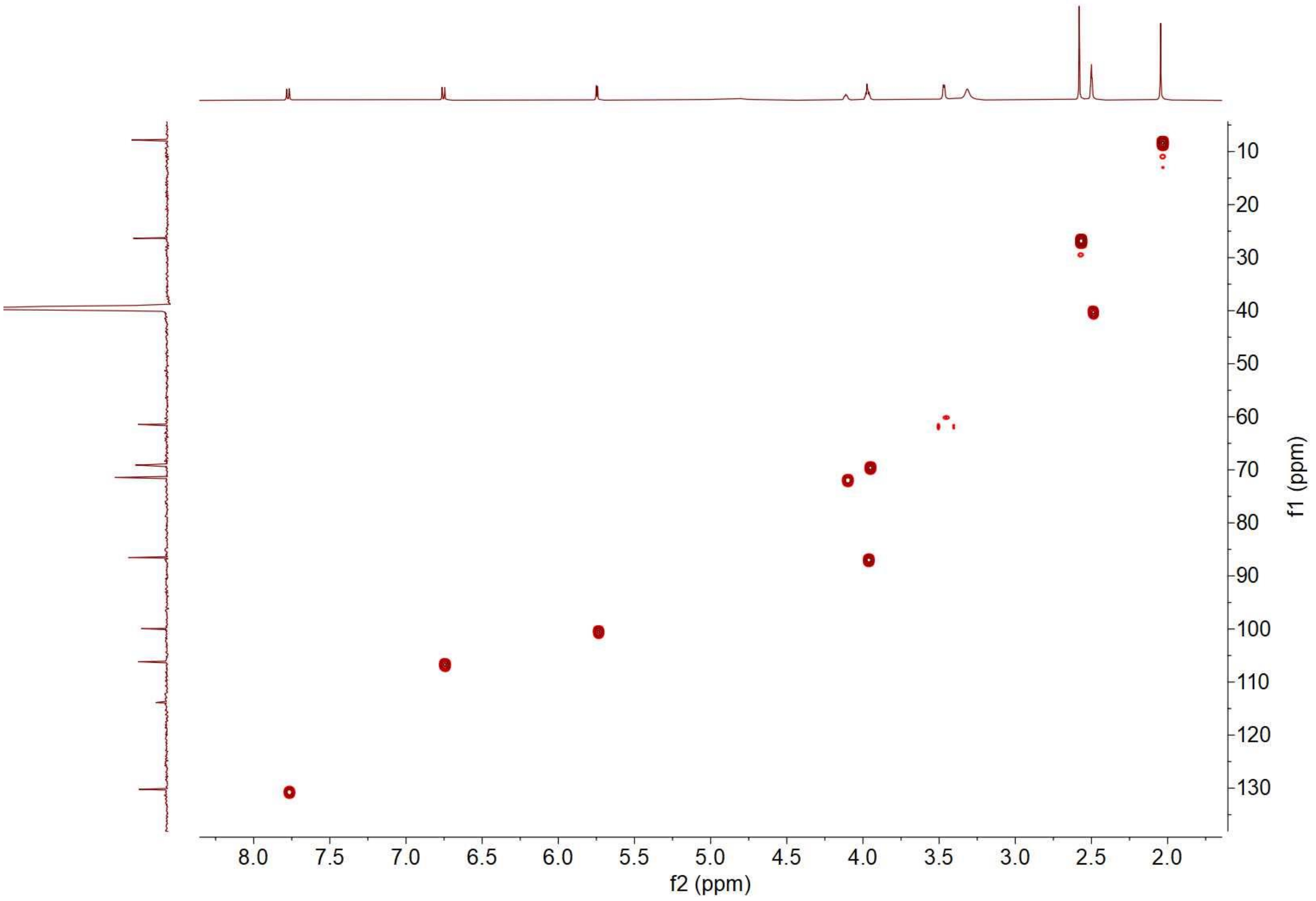

Figure S12. HMBC spectrum of compound **2**;

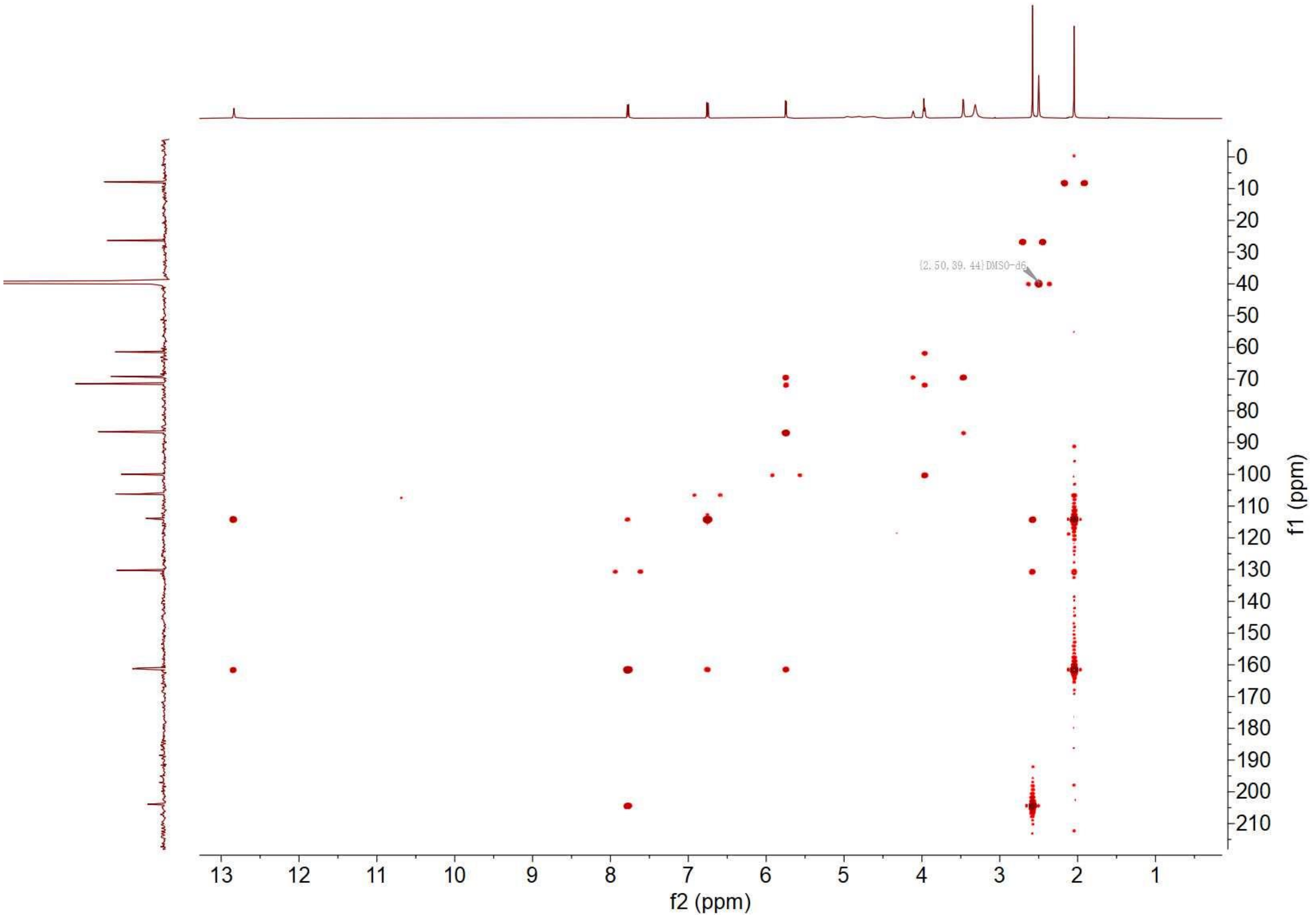

Figure S13. NOESY spectrum of compound **2**;

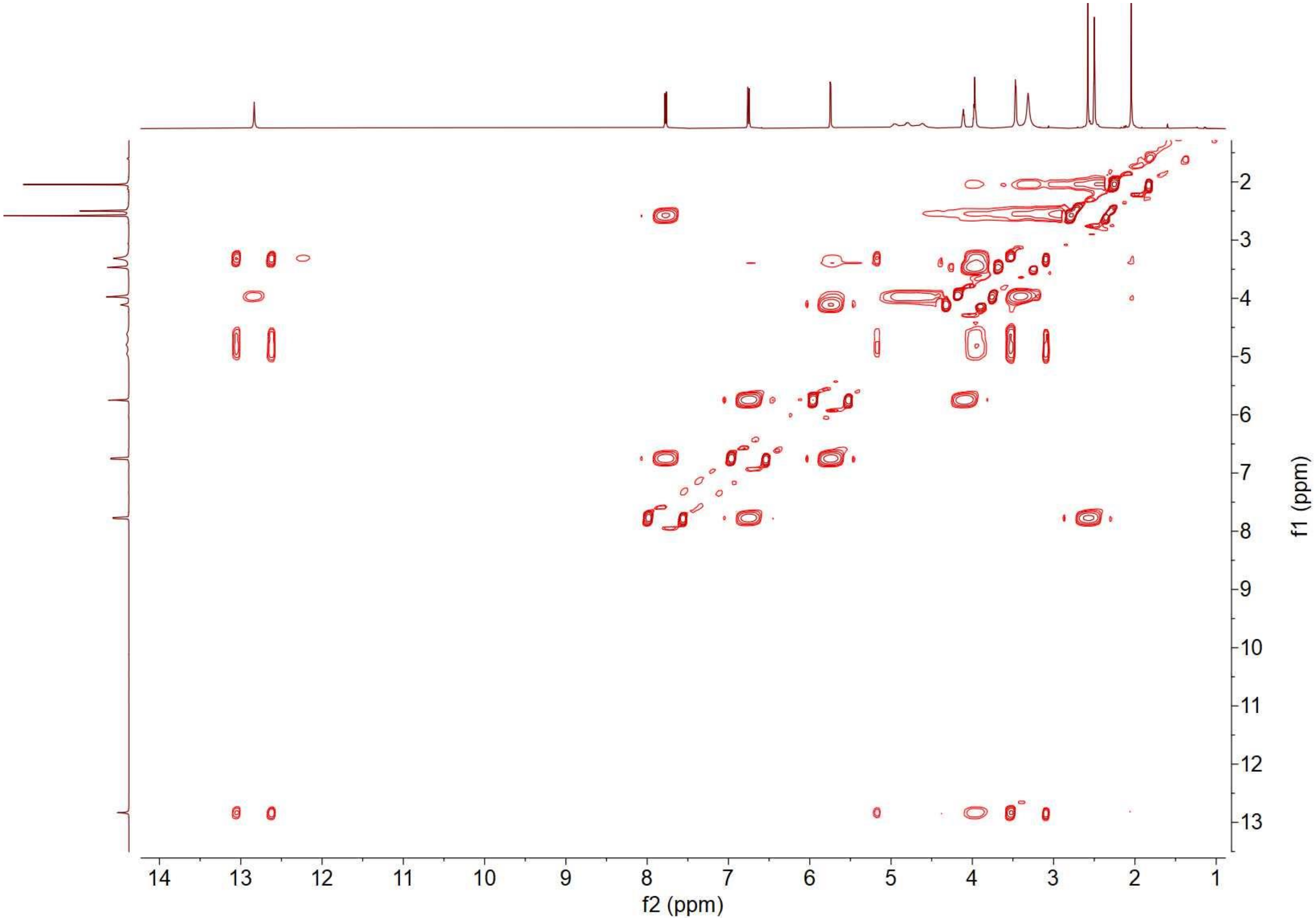

Figure S14. HRESIMS spectrum of compound **2**;

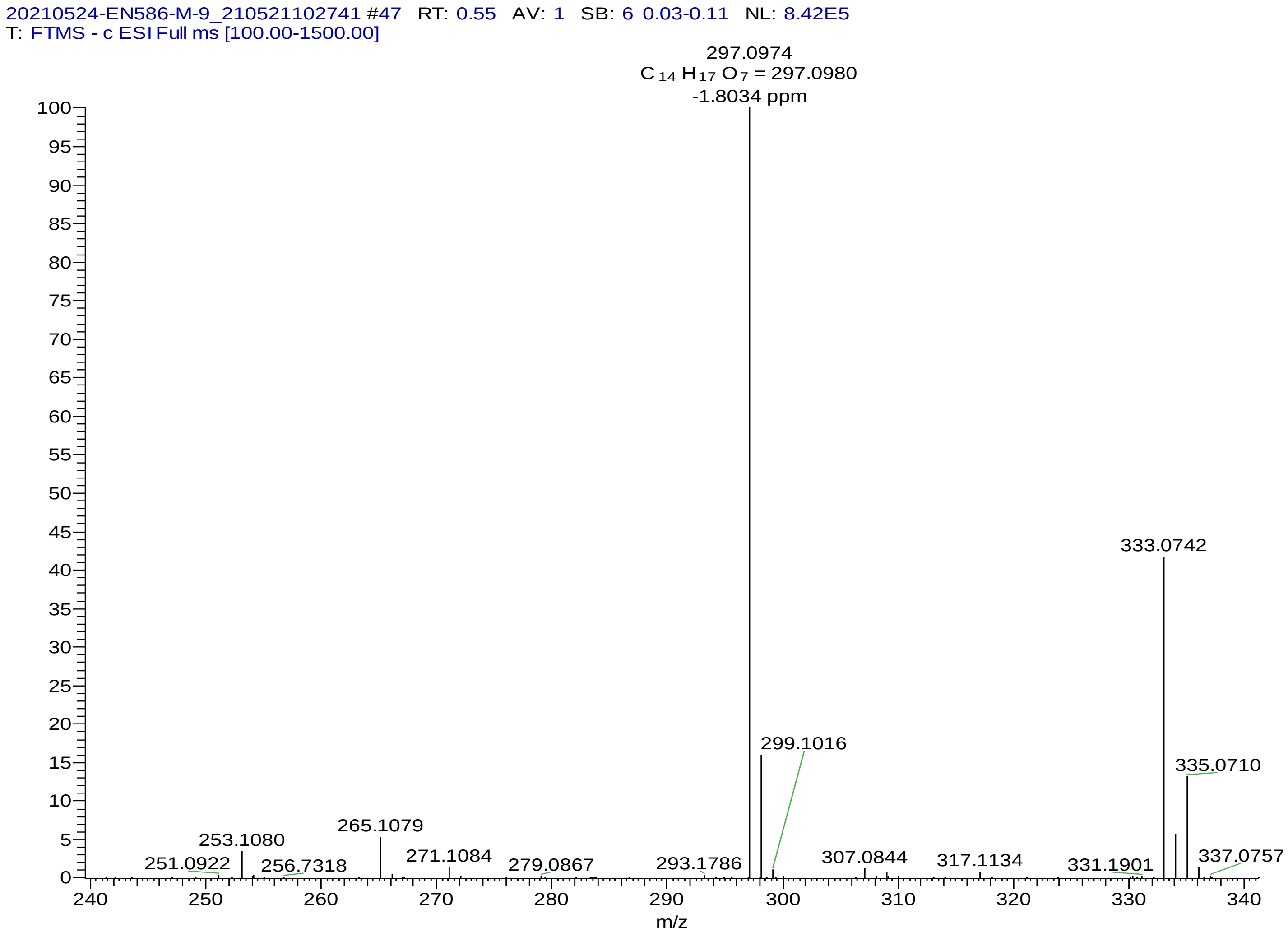

Figure S15. ECD spectrum of compound **2**;

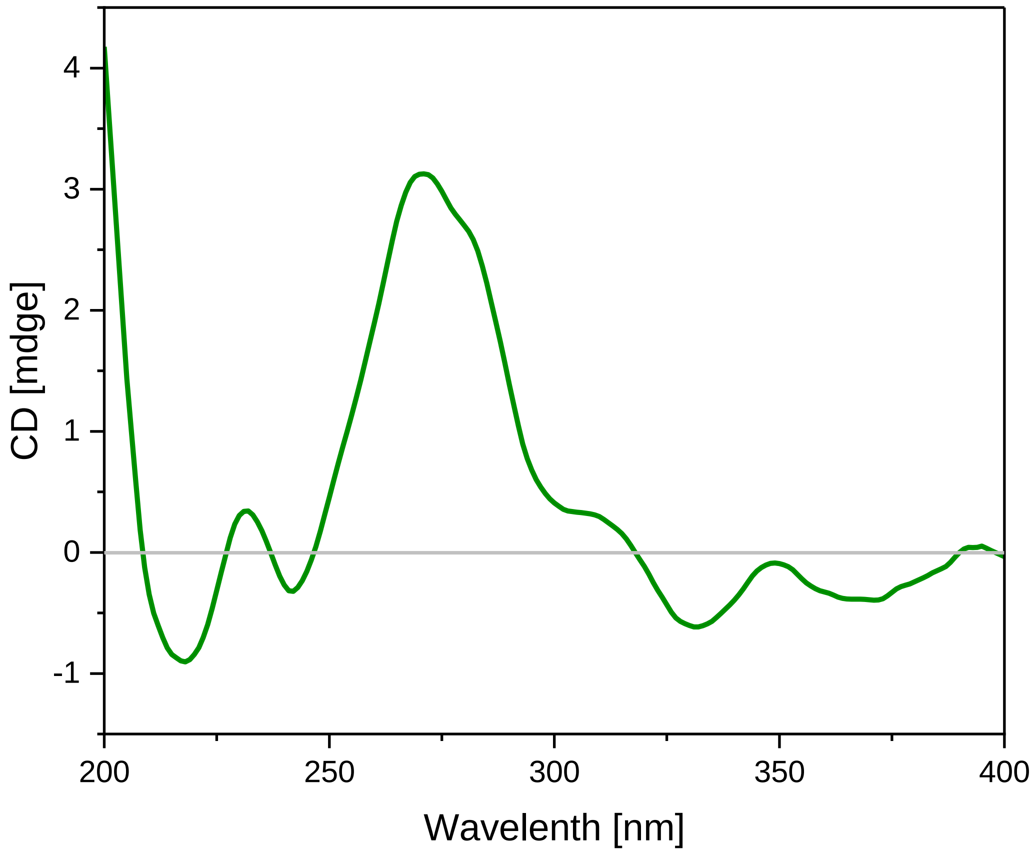

Figure S16. ^{1}H NMR (500 MHz, DMSO-d_6) spectrum of compound **3**;

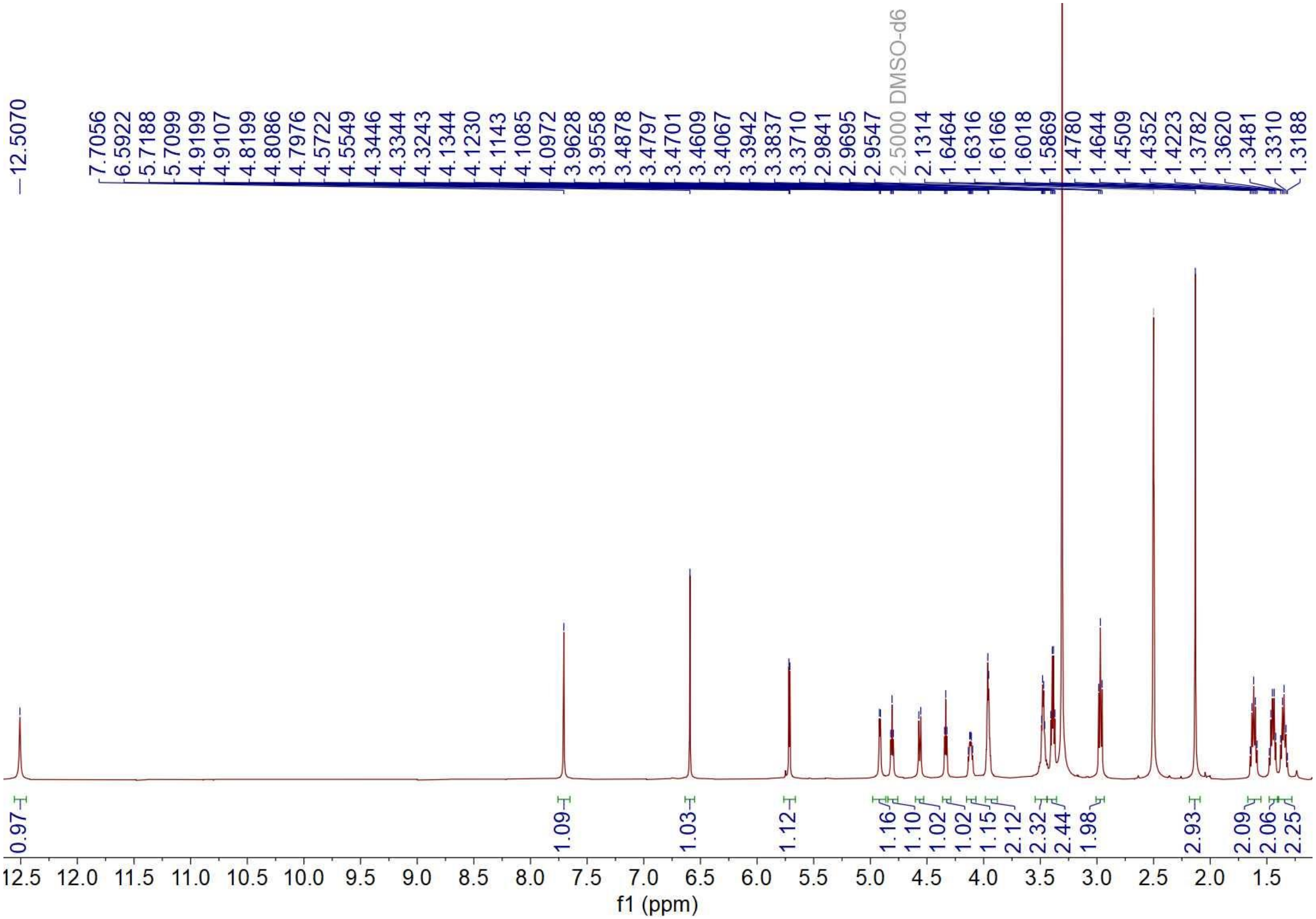

Figure S17. ^{13}C NMR (125 MHz, DMSO-d_6) and DEPT spectra of compound **3**;

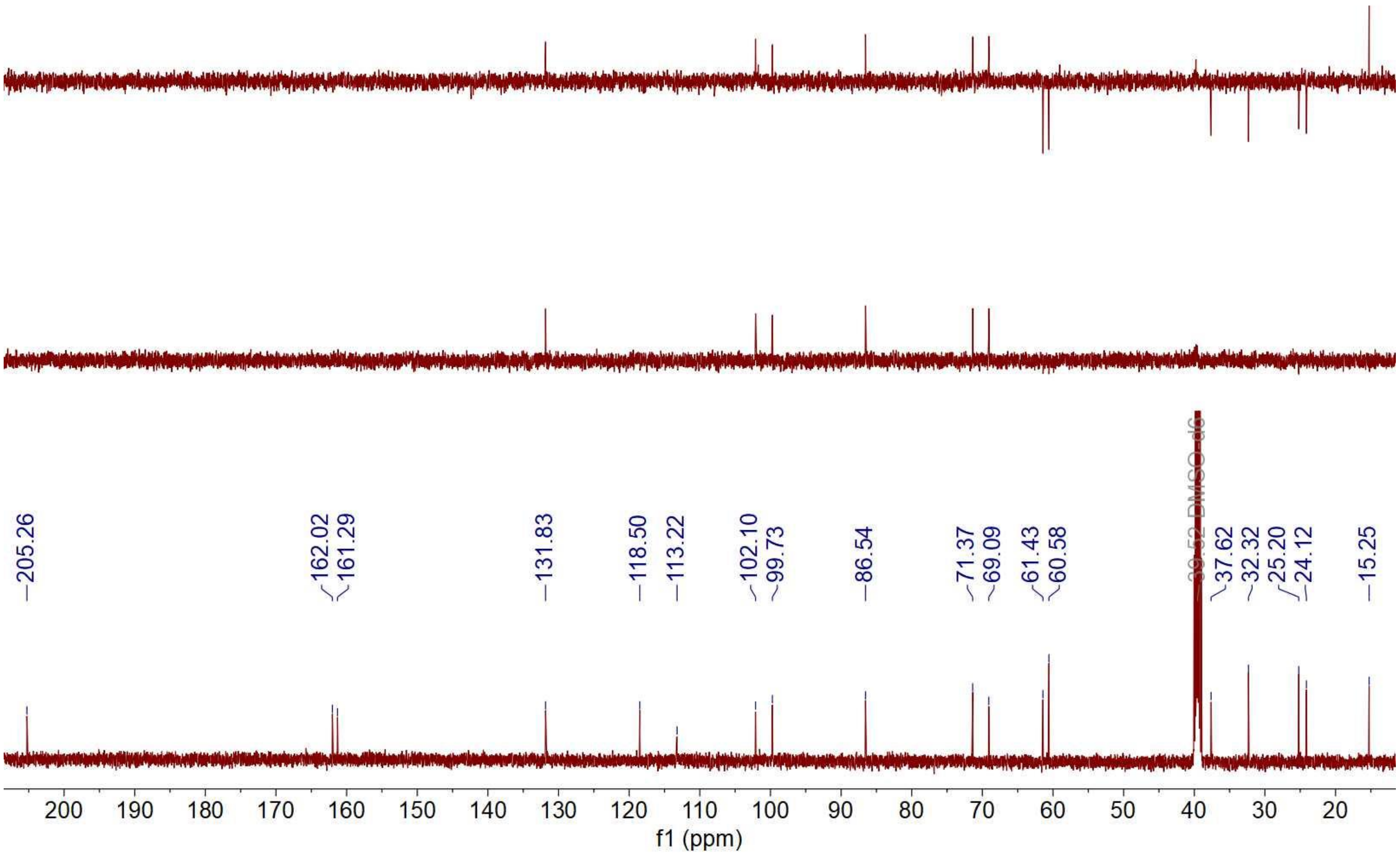

Figure S18. COSY spectrum of compound **3**;

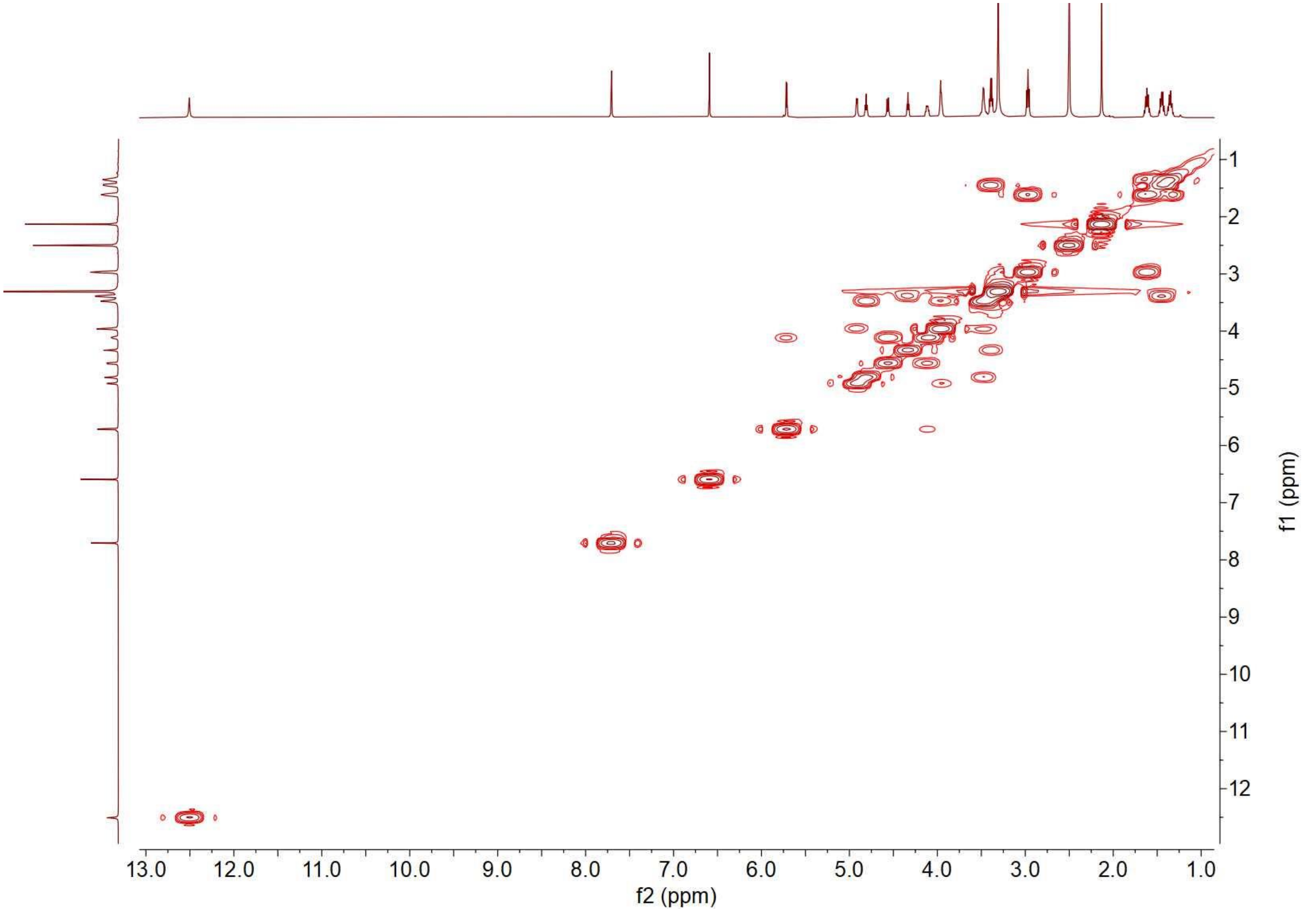

Figure S19. HSQC spectrum of compound **3**;

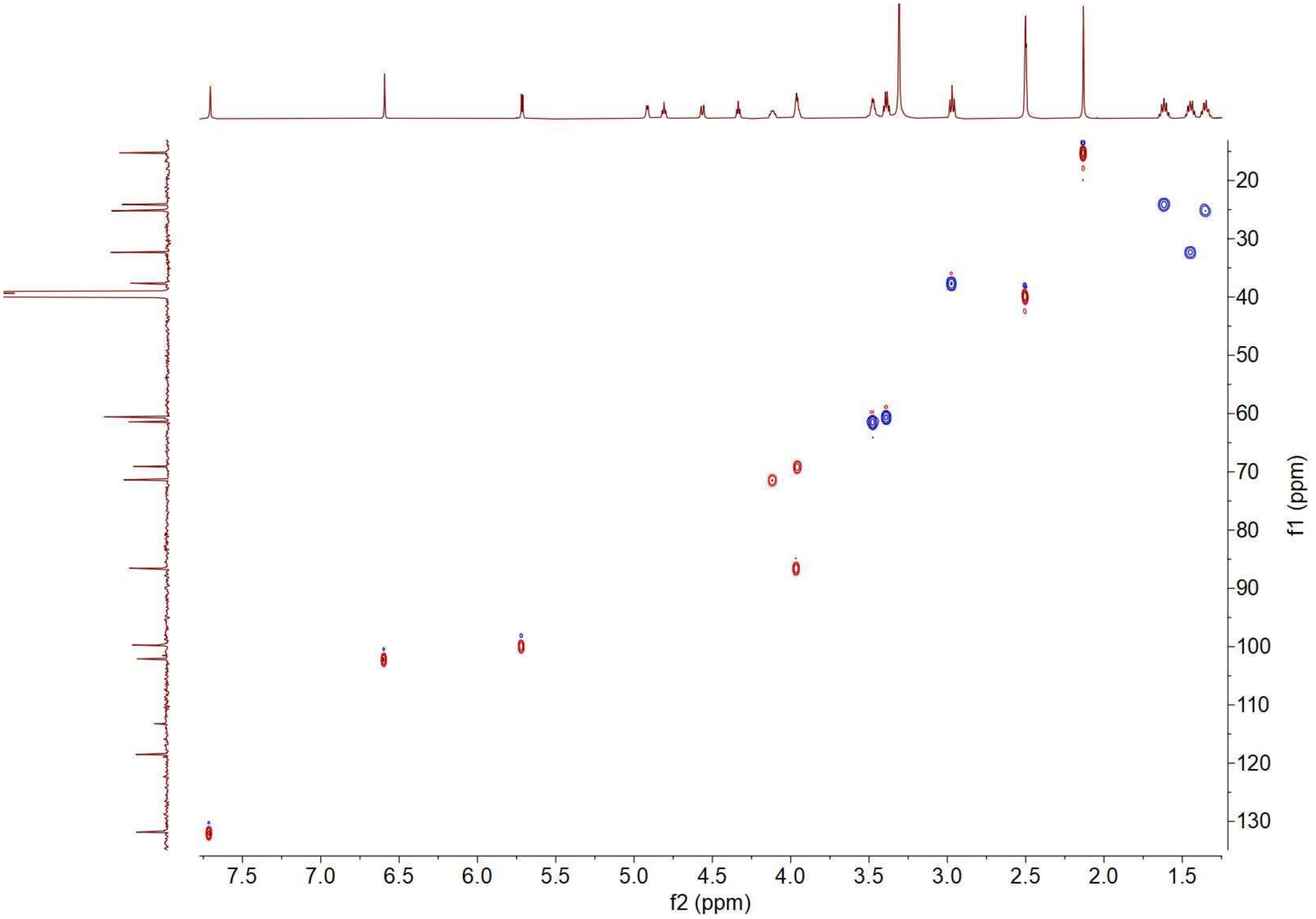

Figure S20. HMBC spectrum of compound **3**;

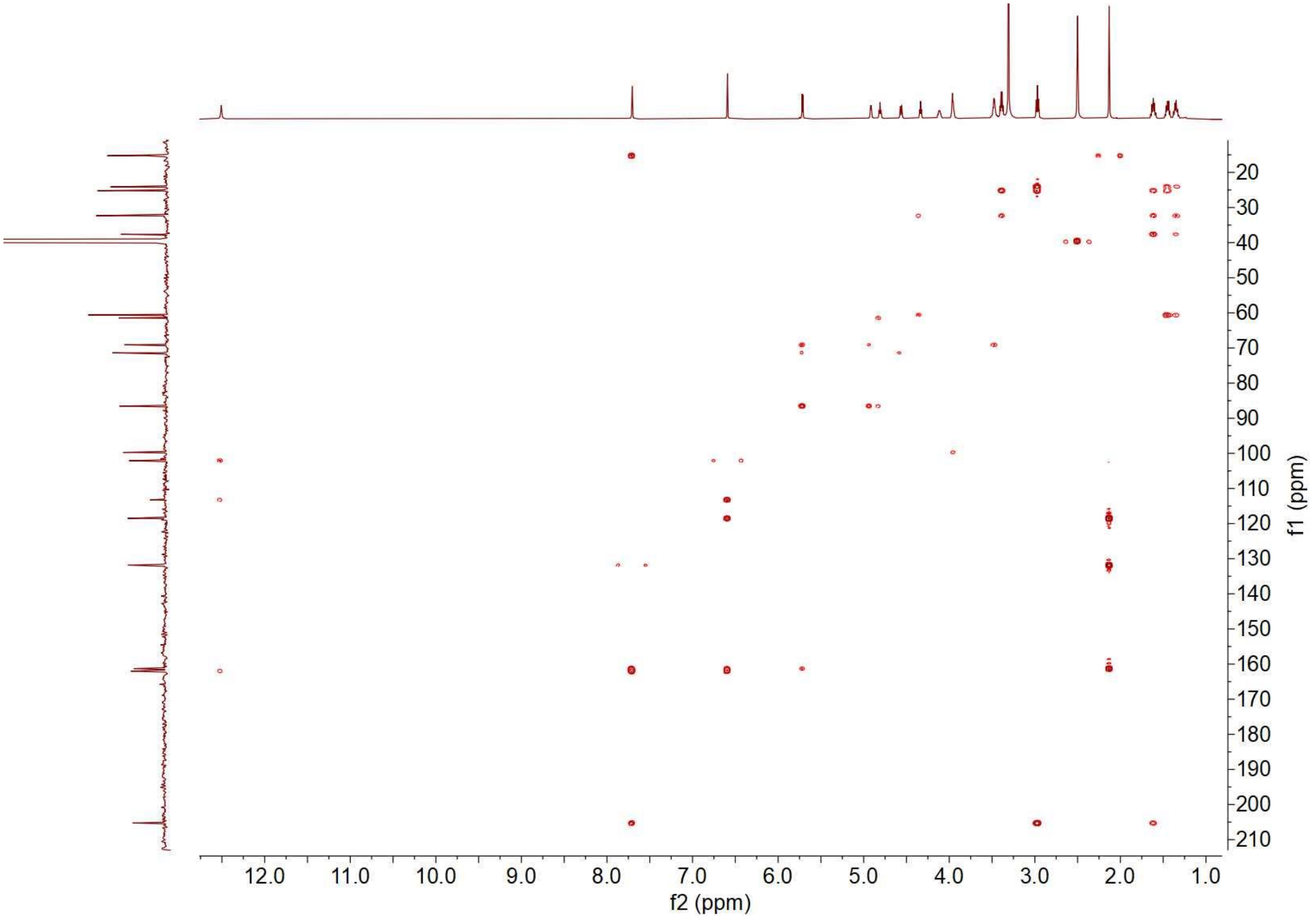

Figure S21. NOESY spectrum of compound **3**;

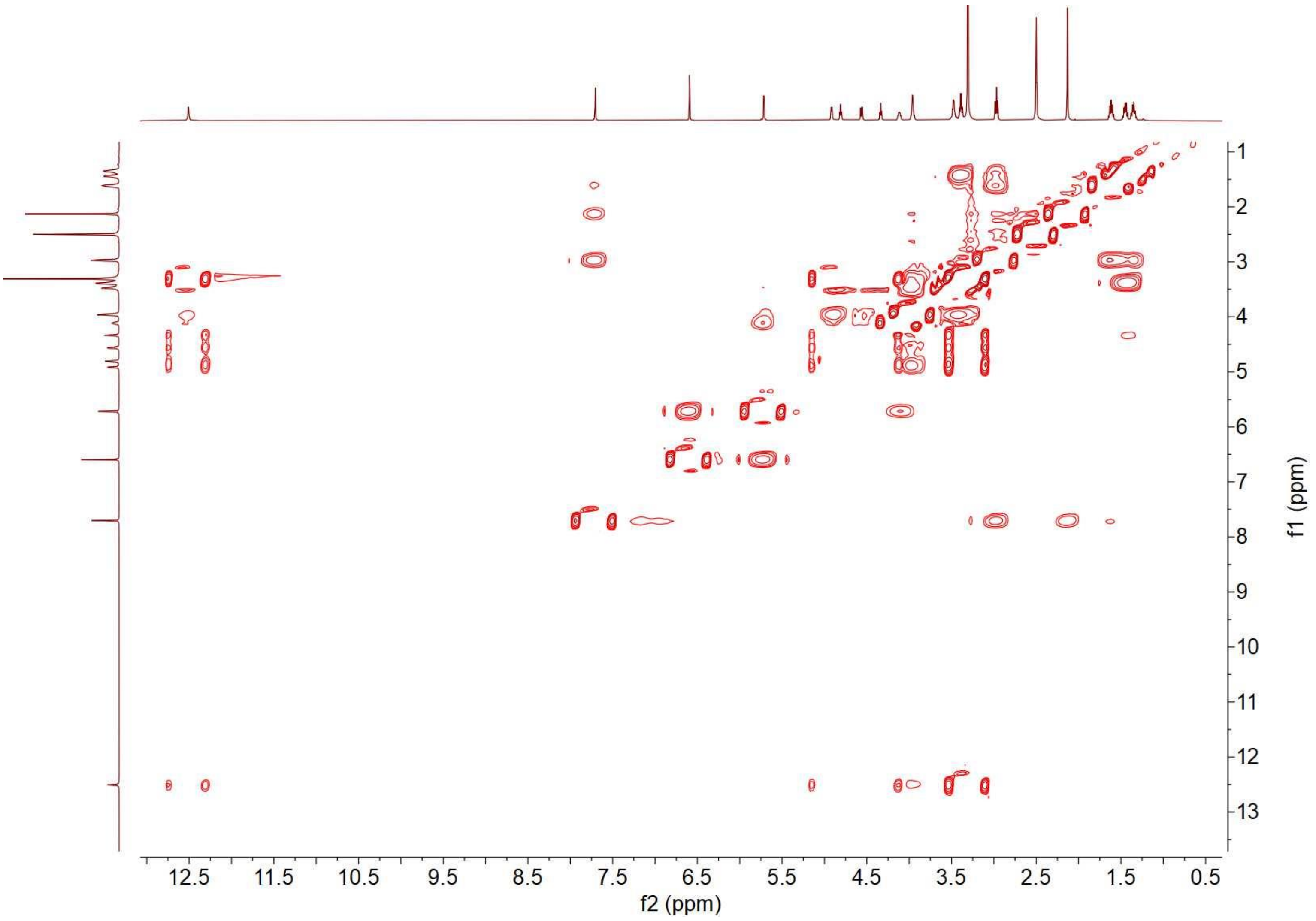

Figure S22. HRESIMS spectrum of compound **3**;

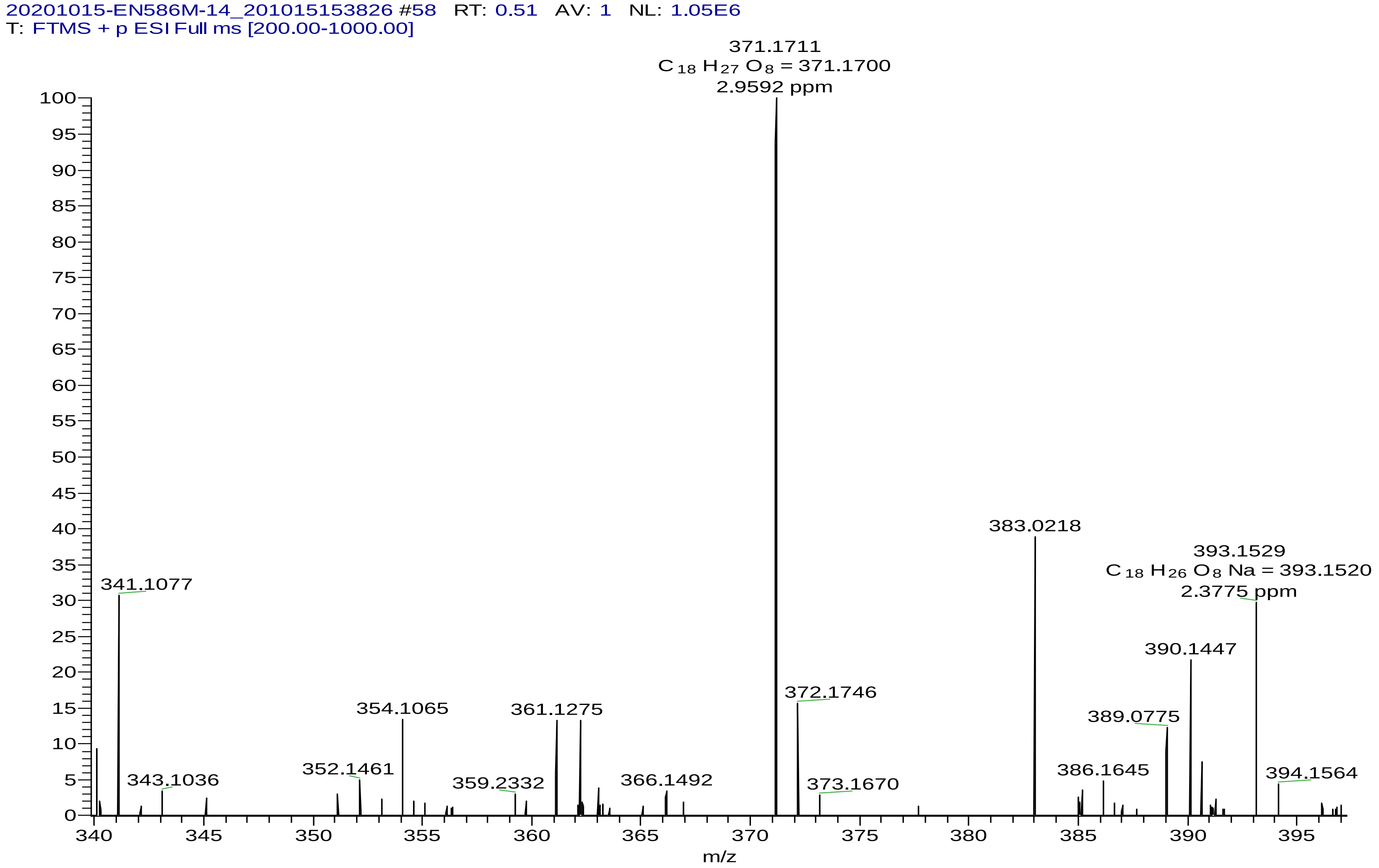

Figure S23. ECD spectrum of compound **3**;

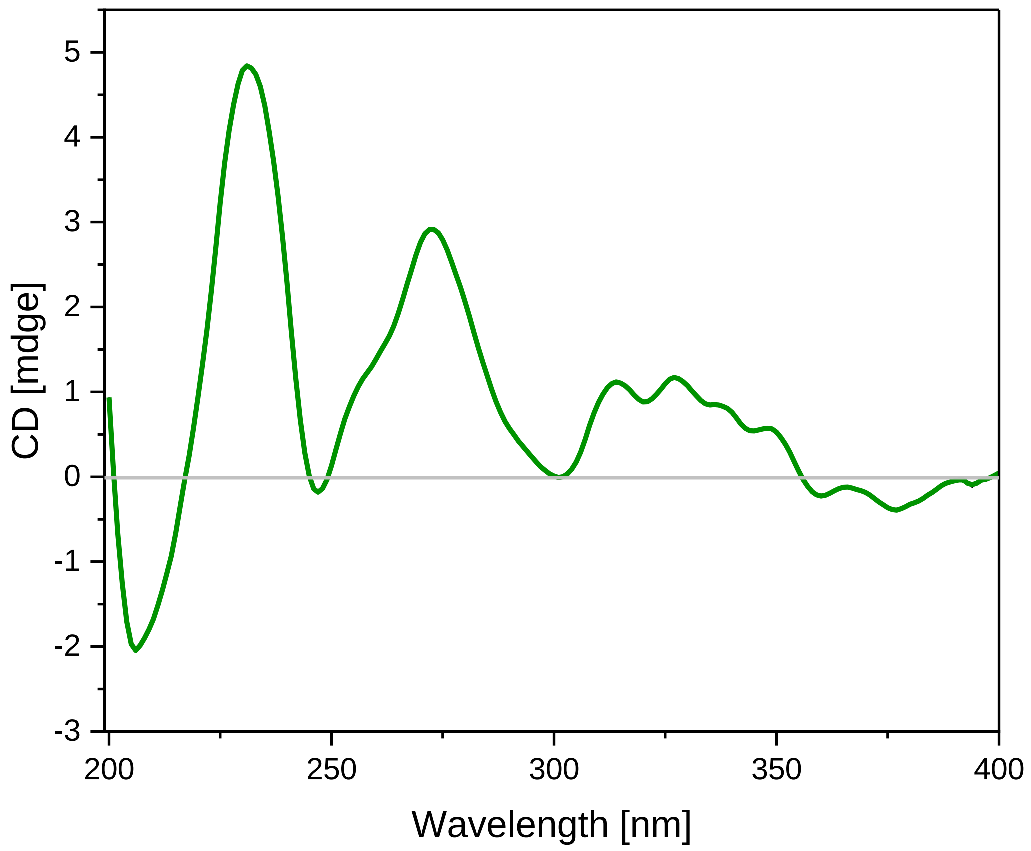

Figure S24. Determination of absolute configuration of glucosamine in compound **1**;

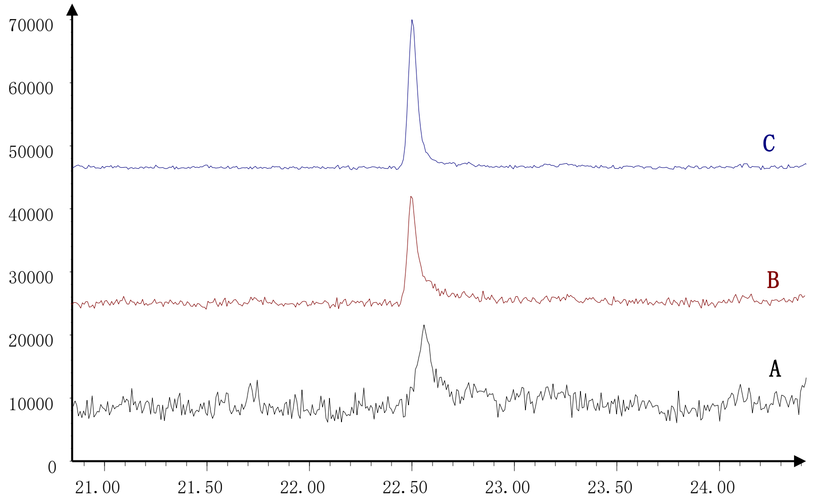

Note: The GC-MS analysis of the glucosamine derivatives (**A**: The derivative of glucosamine in compound **1**; **B**: The mixture of derivatives of glucosamine in compound **1** and authentic D-glucosamine; **C**: The derivative of authentic D-glucosamine).

Figure S25. DP4+ probability Excel sheets of compound **1**;

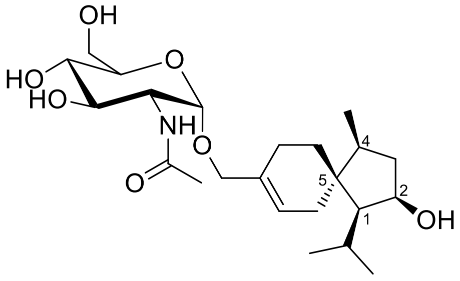

(1*S*,2*R*,4*S*,5*R*)-N-acetyl-α-D-glucosamine

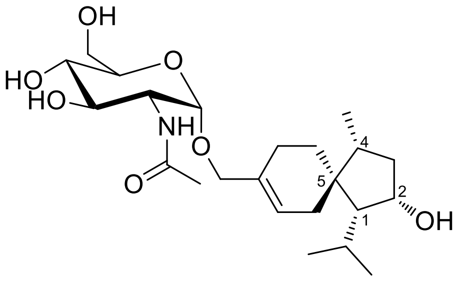

(1*R*,2*S*,4*R*,5*S*)-N-acetyl-α-D-glucosamine

Functional		Solvent?		Basis Set		Type of Data	
mPW1PW91		PCM		6-31+G(d,p)		Shielding Tensors	
		DP4+	0.00%	100.00%	-	-	-
Nuclei	sp2?	xperimenta	Isomer 1	Isomer 2	Isomer 3	Isomer 4	Isomer 5
C	x	169.3	24.3166137	24.4077071			
C	x	137.3	63.5699984	64.9523235			
C	x	124.1	63.8332203	65.076381			
C		96.1	103.651059	102.975603			
C		72.9	118.005321	117.983168			
C		70.8	120.472311	120.962248			
C		70.6	122.056872	121.980299			
C		66.6	122.889942	123.173169			
C		64.6	126.355716	123.474468			
C		60.8	130.646518	130.434294			
C		59.3	135.452393	134.51151			
C		53.9	139.907966	139.62949			
C		46.2	145.415687	144.244513			
C		44.2	147.789776	149.598763			
C		34.5	151.412017	151.366522			
C		31.8	157.26476	156.562598			
C		29.8	167.267305	167.171924			
C		28.5	168.901212	168.31543			
C		26.2	170.087254	169.847795			
C		23.3	171.579151	171.894095			
C		22.8	172.839633	172.63111			
C		22.6	174.208123	174.138205			
C		14.2	179.521987	179.430524			
H		1.24	30.6132119	30.257061			
H		4.22	27.2244562	27.2186282			
H		1.67	29.3771358	29.3333877			
H		1.12	30.2527798	30.1968429			
H		1.55	29.935926	29.9879207			
H		2.07	28.5715588	28.908295			
H		1.79	30.030048	29.6995326			
H	x	5.69	25.2587357	25.3261372			
H		1.71	29.2193065	29.0910729			
H		1.38	29.2550729	29.4906446			
H		1.63	29.4363539	29.5137665			
H		1.19	29.6975581	29.8416783			
H		1.61	29.2099336	29.341043			
H		0.88	30.579176	30.5786512			
H		0.83	30.5527551	30.6690069			
H		0.8	30.3862256	30.5328073			
H		4.08	27.229672	27.1229			
H		4.01	27.4160146	27.1677079			
H		4.65	26.5650957	26.5322584			
H		3.65	27.5649028	27.612099			
H		3.45	27.7145171	27.7041915			
H		3.14	28.1778677	28.2424148			
H		3.39	27.6762096	27.651275			
H		3.6	27.5629579	27.5753457			
H		3.49	27.8202079	27.8261074			
H		1.82	29.5116546	29.5168271			
H		4.54	30.5714255	30.4902428			
H		7.6	25.1202908	25.0864908			
H		4.8	26.8530442	26.7345498			
H		5.03	29.0048145	29.0133332			
H		4.46	29.5367291	29.5526314			

Figure S26. Determination of absolute configuration of ribose in compound **3**;

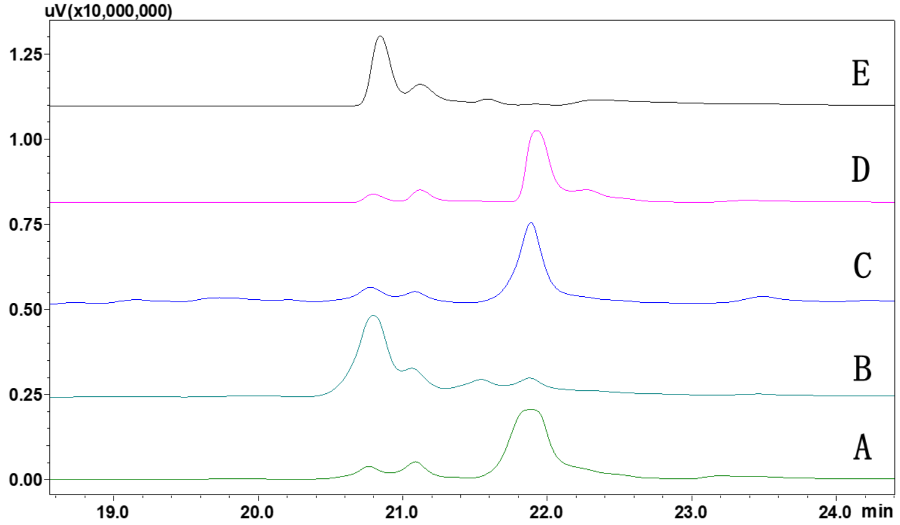

Note: The HPLC analysis of the ribose derivatives (**A**: The mixture of derivatives of the ribose in compound **3** and authentic D-ribose; **B**: The mixture of derivatives of the ribose in compound **3** and authentic L-ribose; **C**: The derivative of the ribose in compound **3**; **D**: The derivative of the authentic D-ribose; **E**: The derivative of the authentic L-ribose)
